# Decoding HuH-7: a comprehensive genetic and molecular portrait of a widely used hepatocellular carcinoma model

**DOI:** 10.3389/fcell.2025.1648639

**Published:** 2025-11-11

**Authors:** Dario Dourado Luis, Thomas Liehr, Stefanie Kankel, Anja Weise, Constanze Pentzold, Eva M. Buhl, Katharina S. Hardt, Diandra T. Keller, Sarah K. Schröder-Lange, Ralf Weiskirchen

**Affiliations:** 1 Institute of Human Genetics, Jena University Hospital, Friedrich Schiller University, Jena, Germany; 2 Electron Microscopy Facility, Institute of Pathology, RWTH University Hospital Aachen, Aachen, Germany; 3 Institute of Molecular Pathobiochemistry, Experimental Gene Therapy and Clinical Chemistry (IFMPEGKC), RWTH, University Hospital Aachen, Aachen, Germany

**Keywords:** molecular profiling, tumorigenesis, drug metabolism, hepatocellular carcinoma, genetic characterization, cell line authentication, transcriptomic analysis, HuH-7

## Abstract

**Introduction:**

Immortalized cell lines play a crucial role in biomedical research by enabling reproducible experiments and enhancing our understanding of complex diseases. HuH-7, originally derived from a human hepatocellular carcinoma, is particularly valuable for studying liver cancer dynamics, viral hepatitis, and drug metabolism. However, concerns about cell line misidentification and genetic drift in cell lines highlight the importance of rigorous authentication to maintain the reliability of research outcomes, despite their widespread use.

**Methods:**

In this study, we present a detailed (cyto)genetic and molecular analysis of HuH-7 cells, focusing on their hepatocellular characteristics and potential applications in translational research. Through thorough genomic profiling and next-generation mRNA expression analyses, we aimed to confirm the authenticity of the cell line and identify key genetic signatures associated with tumorigenic pathways.

**Results and Discussion:**

Our results emphasize the importance of regular identity verification, such as short tandem repeat (STR) profiling, and demonstrate how subtle genetic variations can affect phenotypic traits relevant to modeling liver disease. By providing insights into the genetic and transcriptomic features of HuH-7 cells, this study establishes a robust basis for future research and therapeutic investigations using this widely accepted liver cell model. It also emphasizes the importance for maintaining high-quality standards and robust authentication practices to ensure that cell-based studies produce reliable and reproducible results.

## 1 Introduction

The use of immortalized cell lines is a cornerstone of modern biomedical research, providing reproducible model systems for studying the molecular mechanisms underlying various diseases (Weiskirchen et al., 2023). These cell lines offer several advantages, including infinite proliferative capacity, ease of cultivation, and well-established experimental protocols. These features facilitate large-scale studies and comparative analyses. However, when working with any cell lines, it is crucial to ensure their identity and purity through rigorous authentication to guarantee the validity and reliability of experiments (Souren et al., 2022). Misidentification or cross-contamination of cell lines can lead to inaccurate results and compromise the reproducibility of scientific findings. This highlights the significance of consistently verifying their identity using methods such as short tandem repeat (STR) profiling (Almeida and Korch, 2023).

Among the many cellular models available, the HuH-7 cell line has received particular attention in research focusing on hepatic biology and disease. Established from a human hepatocellular carcinoma (HCC) in 1982, HuH-7 cells exhibit features relevant to the pathological state of liver cancer. This makes them a valuable model for exploring the molecular and genetic underpinnings of tumorigenesis (Nakabayashi et al., 1982). They have been widely used to study viral hepatitis, drug metabolism, cancer development, and liver-specific signaling pathways, providing a platform for *in vitro* high-throughput drug screening and mechanistic analyses (Jiraviriyakul et al., 2025; Li et al., 2025; Zhang et al., 2025). Furthermore, this cell line has played a key role in elucidating the molecular pathways linking chronic liver inflammation and tumor development (Niu et al., 2025).

In the recent years continued refinements in molecular biology and sequencing technologies have emphasized the importance of thoroughly characterizing and authenticating of commonly used cell lines (Horbach and Halffman, 2017; Souren et al., 2022; Weiskirchen et al., 2023). However, awareness and handling of cell misidentification among scientists and journal editorial teams varies widely and often requires significant improvement (Weiskirchen, 2025). Despite the critical importance of accurate cell line identification for the integrity of biomedical research, instances of misidentified or contaminated cell lines persist in published studies. This not only undermines the reproducibility of scientific findings but also poses risks to patient safety when research is translated into clinical practice. Therefore, it is crucial for the scientific community to prioritize rigorous authentication protocols and foster a culture of transparency regarding cell line usage. This will ensure that both researchers and journals actively contribute to maintaining high standards in cellular research.

Detailed genetic and molecular profiling reveals potential differences from the original tumor derived from the patient, helping to mitigate the risk of inaccurate data caused by cell line drift. Therefore, an in-depth understanding of the genetic composition, gene expression profiles, and phenotypic properties of HuH-7 are of considerable interest to the broader scientific community. The karyotype of the HuH-7 line is known to be severely abnormal (Ding et al., 2013). Furthermore, a previous study using single-cell analysis, multi-color fluorescence *in situ* hybridization (M-FISH), single nucleotide polymorphism (SNP) microarrays, and amplicon sequencing has already defined a reference genome profile for HuH-7. This study revealed that the HuH-7 cell line exhibits complex chromosomal abnormalities affecting all chromosomes, as well as substantial loss of heterozygosity (Kasai et al., 2018). This previous study has suggested that the HuH-7 is a highly heterogeneous population. Therefore, it is mandatory to re-examine the HuH-7 cell line after all these years to ensure its continued validity as a model for research.

This study aims to extend the comprehensive genetic and molecular characterization of the HuH-7 cell line, with a focus on verifying its identity and identifying features that are unique to HCC. This will enhance the reliability and biological relevance of future research utilizing HuH-7 cells and contribute to more robust and reproducible findings in liver disease research. Furthermore, by comparing our genetic data with those of previous studies from other laboratories, we intend to provide an approximate estimate of the heterogeneity of this tumor cell line. As such, the present work was conceived as a resource-building study. Rather than focusing on a specific pathogenic mechanism in animals, our goal was to document the stable and variable characteristics of low-passage HuH-7 cultures at the chromosome, RNA, and protein levels. This ‘molecular portrait’ serves as a reference point for individual laboratories to assess their own HuH-7 stocks and provides a strong foundation for future mechanistic studies, such as xenograft models, where well-documented starting materials are essential.

## 2 Materials and methods

### 2.1 Literature research

A literature search was conducted on 17 May 2025 in PubMed (National Center for Biotechnology Information, 2025) to identify publications involving Huh-7 cells. The search term “Huh7 or Huh-7” was used to encompass all relevant papers, regardless of minor variations in cell line nomenclature. The retrieved articles were then assessed for their relevance to our study, as well as for information on experimental techniques, results, and applications involving HuH-7 cells.

### 2.2 Cell culture

The human cell line HuH-7 (#JCRB0403, RRID: CVCL_0336) was obtained from the Japanese Collection of Research Bioresources (JCRB) via the FUJIFILM Wako Chemicals Europe GmbH representative. The cells were cultured in a humidified incubator at 37 °C with 5% CO_2_ in Dulbecco’s Modified Eagle Medium (DMEM, high glucose #6171, Sigma-Aldrich, Merck, Taufkirchen, Germany) with 1.5 g/L sodium bicarbonate. This medium was supplemented with 10% fetal bovine serum (FBS, #F7524, Sigma-Aldrich), 4 mM L-glutamine (#G7513, Sigma-Aldrich), and 1× penicillin-streptomycin antibiotic solution (DE17-602E, Lonza, Cologne, Germany). The cells were passaged when they reached 80%–90% confluence, using an Accutase solution (A6964-100 ML, Sigma-Aldrich), and seeded at the appropriate density for subsequent analyses. All experiments were conducted using cells from low passage numbers to minimize phenotypic drift.

### 2.3 Short tandem repeat profiling and *Mycoplasma* testing

To confirm the authenticity of the HuH-7 cell line and identify any *Mycoplasma* spp. contamination, we performed short tandem repeat (STR) profiling and a sensitive real-time PCR assay to detect even trace amounts of the bacterium. For this purpose, we utilized the cell line authentication service provided by IDEXX (Kornwestheim, Germany) through the CellCheck™ 16 Human PLUS (test code: 42–00098). This test system includes the 13 human reference STR loci recommended for human cell line authentication: CSF1PO, D3S1358, D5S818, D7S820, D8S1179, D13S317, D16S539, D18S51, D21S11, FGA, TH01, TPOX, and vWA (Almeida and Korch, 2023; Korch et al., 2021), two additional variant markers (Penta D and Penta E), and the amelogenin (AMEL) locus, which is used to determine gender. Additionally, we conducted regular *mycoplasma* testing in our laboratory. Cell culture supernatants from 80%–90% confluent cultures were directly processed with the Venor®GeM OneStep kit for conventional PCR (#11–8050, Minerva Biolabs GmbH, Berlin, Germany). The resulting amplicons were analyzed on a 2% standard agarose gel and stained with Midori Green, in accordance with an established standard protocol (Supplementary Figure S1) (Weiskirchen et al., 2023).

### 2.4 Western blot analysis

For the protein expression studies, cells were lysed in an ice-cold RIPA buffer supplemented with protease and phosphatase inhibitors. Protein concentration was determined using the DC Protein Assay (#5000116, Bio-Rad Laboratories, CA, USA). Subsequently, 60 µg of cell lysate was separated using sodium dodecyl sulfate-polyacrylamide gel electrophoresis (SDS-PAGE), as previously described (Liehr et al., 2025). The proteins were then transferred onto 0.45 µm nitrocellulose membranes, blocked with a 5% non-fat dry milk solution, and incubated overnight at 4 °C with primary antibodies specific to the targets of interest. After washing, the membranes were incubated with the relevant secondary antibodies conjugated to horseradish peroxidase (HRP). Protein bands were visualized using the Supersignal™ West Dura Extended duration Substrate (#34076, Thermo Fisher Scientific, Schwerte, Germany). Information on the antibodies, including their RRID identifiers, used in this study is provided in Table 1. In the Western blot analysis we used protein extracts generated from human livers as controls. These were obtained from the centralized RWTH Biomaterial Bank (https://www.ukaachen.de/kliniken-institute/institut-fuer-pathologie/biobank/) with approval from the Ethics Committee of the Medical Faculty of RWTH Aachen University (permit number EK 206/09). Human liver tissue was homogenized in a MM400 mixer mill (Retsch GmbH, Haan, Germany) using established protocols (Liehr et al., 2025). Following protein quantification, 80 µg of protein tissue lysate was utilized for Western blot analysis.

**TABLE 1:** Antibodies used for Western blot analysis.

Antibody	Cat.-No	RRID^a^	Company	Dilution	Clonality
AFP	MIA1301	AB_11153904	Invitrogen	1:1,000	m mAb
Albumin	#4929	AB_2225785	Cell Signaling	1:1,000	r pAb
Arginase 1	16001-1-AP	AB_2289842	Proteintech	1:1,000	r pAb
Collagen I	14695-1-AP	AB_2082037	Proteintech	1:1,000	r pAb
Collagen III	22734-1-AP	AB_2879158	Proteintech	1:1,000	r pAb
Cyclophilin A (PPIA)	#2175	AB_2169116	Cell Signaling	1:1,000	r pAb
CYP3A4	18227-1-AP	AB_2090329	Proteintech	1:1,000	g pAb
Cytokeratin 19	SAB5600252	AB_3717691	Sigma-Aldrich	1:1,000	r mAb
FABP1	13626-1-AP	AB_2102017	Proteintech	1:1,000	r pAb
Ferritin heavy chain (FTH)	#4393	AB_11217441	Cell Signaling	1:1,000	r mAb
Ferritin light chain (FTL)	ab69090	AB_1523609	Abcam	1:1,000	r pAb
Fibronectin	AB1954	AB_11213226	Millipore	1:1,000	r pAb
GAPDH (6C5)	sc-32233	AB_627679	Santa Cruz Biotech	1:1,000	m mAb
HNF4α^b^	sc-6556	AB_2117025	Santa Cruz	1:1,000	g pAb
p53	OP43	AB_564964	Millipore	1:1,000	m mAb
Transferrin	17435-1-AP	AB_2035023	Proteintech	1:1,000	r pAb
α-2-Macroglobulin	200–101-207	AB_2610865	Rockland	1:1,000	g pAb
α-SMA	CBL171-I	AB_3076220	Sigma-Aldrich	1:1,000	m mAb
α-Tubulin (B-7)	sc-5286	AB_628411	Santa Cruz Biotech	1:1,000	m mAb
β-Actin	A5441	AB_476744	Sigma-Aldrich	1:10,000	m mAb
Goat anti-rabbit IgG (H + L), HRP	#31460	AB_228341	Invitrogen	1:5,000	g pAb
Goat anti-mouse IgG (H + L), HRP	#31430	AB_228307	Invitrogen	1:5,000	g pAb
Mouse anti-goat IgG (H + L), HRP	#31400	AB_228370	Invitrogen	1:5,000	m pAb

^a^ Data were taken from the Research Resource Identifier (RRID) portal, which is available at https://www.rrids.org/. Abbreviations used: g, goat; HRP, horse-radish peroxidase; m, mouse; mAb, monoclonal antibody. pAb, polyclonal antibody; r, rabbit.

^b^ Distribution of this antibody has been discontinued.

### 2.5 Combined karyotype and M-FISH analysis and multicolor banding

For the combined karyotype and M-FISH analysis, metaphase chromosome spreads were prepared by treating HuH-7 cells with colcemid to arrest them in metaphase, following the detailed protocol previously described (Liehr et al., 2024). After hypotonic treatment and fixation in methanol-acetic acid, the chromosome spreads were placed dropwise onto glass slides and hybridized with a M-FISH probe mixture containing 24 whole chromosome painting probes specific to the 24 different human chromosomes (#D-0125–120-DI, XCyting, MetaSystems Probes, Altlussheim, Germany). Chromosome banding was performed using inverted 4′,6-diamidino-2-phenylindole (DAPI, #D1306, Thermo Fisher Scientific) staining; 20 metaphases were analyzed in detail. We analyzed karyotypes across all 20 metaphases observed by mFISH using whole chromosome paints and also confirmed and refined the results with 20 chromosome-specific multicolor banding (MCB) experiments. For each MCB experiment 20–25 metaphases were analyzed. Additionally, 24 homemade chromosome-specific MCB probe sets were applied as previously described (Weise et al., 2008), and 20–25 metaphases were analyzed for each. All FISH analyses were conducted using a Zeiss Axioplan fluorescence microscope (Carl Zeiss Jena, Jena, Germany) equipped with a CCD camera and image processing software (ISIS, MetaSystems, Altlussheim, Germany.

### 2.6 Array comparative genomic hybridization

The losses and gains of chromosomal regions in human chromosomes were identified using chromosomal microarray as array comparative genomic hybridization (aCGH). DNA was isolated from the cell line and aCGH analysis was performed using 4 × 180 K SurePrint G3 Human CGH Microarray slides (Agilent Technologies). These slides cover the entire human genome with a 13-kb overallmedian probe spacing (11 kb in refseq genes). The probes were combined with reference DNA (male) provided by Promega as controls and processed as previously reported in GRCh37/hg19 (Aust et al., 2013). The data obtained was compared with CGH-results obtained in human hepatocellular carcinoma (Guan et al., 2000).

### 2.7 Next-generation sequencing (NGS) mRNA bulk sequencing

For the transcriptomic analysis, total RNA was extracted from HuH-7 cells that had been grown to 80% confluency using an established CsCl density gradient protocol (Liehr et al., 2024). Quantity and quality/integrity were evaluated via UV spectroscopy and on the Agilent 4200 TapeStation instrument (Agilent Technologies Inc., Waldbronn, Germany). Library preparation involved mRNA enrichment or rRNA depletion, followed by fragmentation, synthesis of first- and second-strand cDNA synthesis, adapter ligation, and PCR amplification according to established protocols (Liehr et al., 2025). The libraries were then sequenced on a high-throughput Illumina sequencing platform with prefilled cartridges and the downstream analysis was performed using standardized pipelines at the IZKF Genomic Facility of the University Hospital Aachen. The obtained sequence data were aligned to the human reference genome (Genome assembly GRCh38: https://www.ncbi.nlm.nih.gov/datasets/genome/GCF_000001405.26/), and quantified at the gene and transcript levels. This resulted in length-normalized transcripts per million (TPM) values.

### 2.8 Microscopic analysis

Cellular morphology was routinely monitored using phase-contrast or bright-field microscopy to assess growth patterns and overall health. Images were captured using a Leica EC3 digital camera connected to a Leica DM IL LED microscope fitted with the Leica Application Suite (LAS) software (version 3.4.0, Leica Microsystems GmbH, Wetzlar, Germany).

### 2.9 Phalloidin staining

For Phalloidin Staining, 30,000 cells were seeded in four-chamber culture sides (#354104, BD Falcon™, BD Biosciences, Erembodegem, Belgium). After 72 h, microfilament staining was conducted as previously described (Liehr et al., 2025). The cells were stained with a 1× diluted Rhodamine-Phalloidin solution (#R415, Invitrogen, Thermo Fisher Scientific, Schwerte, Germany) for 20 min in the dark. Nuclear counterstain was performed with 4′,6-diamidino-2-phenylindole (DAPI, #D1306, Thermo Fisher Scientific) for 30 min in the dark. After staining, the slides were embedded in Vectashield Antifade mounting medium (#H-1000, Burlingame, California, United States) and stored at 4 °C. For analysis, a Nikon Eclipse E80i fluorescence microscope with NIS-Elements Vis software (version 3.22.01) was used.

### 2.10 Electron microscopy analysis

For ultrastructure studies, the cells were harvested and fixed in a solution of 2.5% glutaraldehyde solution in a 0.1 M phosphate buffer. They were then post-fixed in 1% osmium tetroxide. Following dehydration using a graded ethanol series, the samples were embedded in epoxy resin. Ultrathin sections (approximately 70–90 nm thick) were cut using an ultramicrotome and mounted on copper grids. These sections were subsequently post-stained with uranyl acetate and lead citrate prior to examination using a Zeiss Leo 906 transmission electron microscope (Carl Zeiss AG, Oberkochen, Germany) operating at 60 kV. High-resolution micrographs were taken at various magnifications ranging from ×2,784 to 12,930× in order to elucidate the organelle integrity of organelles and the morphology of mitochondria and other subcellular features that are characteristic of HuH-7 cells.

## 3 Results

### 3.1 Usage of HuH-7 cells in biomedical research

The Huh-7 cell line was first established in 1982 by Hidekazu Nakabayashi and his team at Kagawa University. It originated from a well-differentiated hepatocyte carcinoma that was removed during surgery from a 57-year-old Japanese male (Nakabayashi et al., 1982). A PubMed search conducted on 17 May 2025 using the search term “HuH-7 or HuH7” revealed that this cell line has been used in 8,319 studies. Furthermore, HuH-7 cells have been referenced over 5,000 times in the RRID portal (https://rrid.site/data/source/SCR_013869-1/search?q=CVCL_0336&l=CVCL_0336), which underscores the extensive use of this cell line in biomedical research.

### 3.2 Appearance of HuH-7 as assessed by light microscopy

**FIGURE 1:**
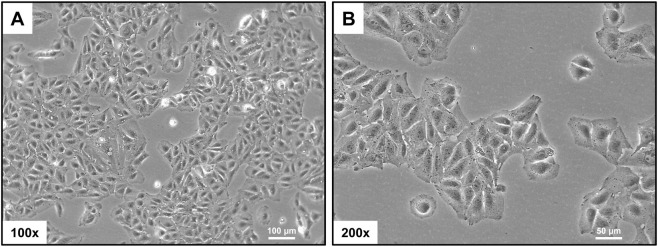
Morphological assessment. Phase-contrast micrographs show the characteristic polygonal shape and epithelial-like growth pattern of HuH-7 cells. The scale bar indicates 100 µm in panel **(A)** and 50 µm in panel **(B)**.

When observed under a light microscope, HuH-7 cells exhibit an epithelial morphology. They grow as adherent, polygonal cells with a relatively uniform appearance (Figure 1). They often form monolayers in culture, with rounded or oval nuclei that have prominent nucleoli and abundant cytoplasm. Binucleation and characteristic intercellular contacts may occasionally be observed. These morphological features are consistent with the cells being derived from a well-differentiated hepatocellular carcinoma tissue.

### 3.3 Short tandem repeat profiling for HuH-7

**FIGURE 2:**
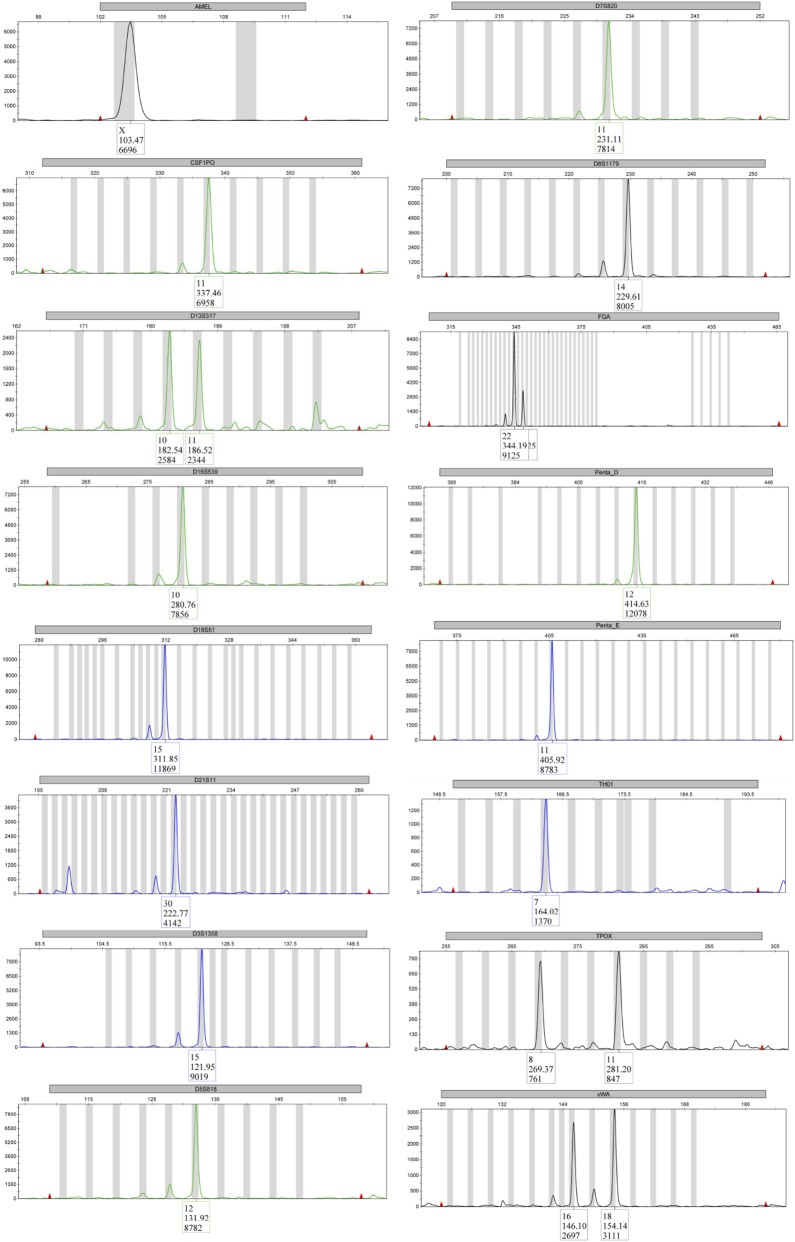
STR profiling. This representative electropherogram shows the amplified STR loci for HuH-7. The unique peaks confirm the identity of the cell line when compared with reference databases.

For authentication purposes, we performed short tandem repeat (STR) profiling on our Huh-7 cells. We analyzed 15 specific STR markers, including Penta E, D18S51, D21S11, TH01, D3S1358, FGA, TPOX, D8S1179, vWA, Penta D, CSF1PO, D16S539, D7S820, D13S317, and D5S818, as well as the amelogenin gene to determine sex (Figure 2; Table 2). The resulting profile showed a 100% match to the published reference data (Kasai et al., 2018), thus confirming the authenticity and genetic consistency of our HuH-7 cell line.

**TABLE 2:** Short tandem repeat (STR) profiling of HUH-7 cells using 15 human-specific STR loci and the amelogenin locus.

Marker name	Cytogenetic location^a^	Repeat sequence pattern (5’→3′)^a^ ^,^ ^b^	Sample results	Reference^c^
AMEL	Xp22.1–22.3 and Y	NA	X	X
CSF1PO	5q32	[ATCT]_n_	11	11
D13S317	13q31.1	[TATC]_n_	10, 11	10, 11
D16S539	16q24.1	[GATA]_n_	10	10
D18S51	18q21.33	[AGAA]_n_	15	15
D21S11	21q21.1	[TCTA]_n_ [TCTG]_n_ [TCTA]_n_ TA [TCTA]_n_ TCA [TCTA]_n_ TCCATA [TCTA]n	30	30
D3S1358	3p21.31	[TCTA]_n_ [TCTG]_n_ [TCTA]_n_	15	15
D5S818	5q23.2	[ATCT]_n_	12	12
D7S820	7q21.11	[TATC]_n_	11	11
D8S1179	8q24.13	[TCTA]_n_ [TCTG]_n_ [TCTA]_n_	14	14
FGA	4q31.3	[GGAA]_n_ [GGAG]_n_ [AAAG]_n_ [AGAA]_n_ [AAAA]_n_ [GAAA]_n_	22, 23	22, 23
Penta_D	21q22.3	[AAAGA]_n_	12	12
Penta_E	15q26.2	[TCTTT]_n_	11	11
TH01	11p15.5	[AATG]_n_	7	7
TPOX	2p25.3	[AATG]_n_	8, 11	8, 11
vWA	12p13.31	[TAGA]_n_ [CAGA]_n_ [TAGA]_n_	16, 18	16, 18

^a^ Data depicted was retrieved from the Short Tandem Repeat DNA Database (https://strbase.nist.gov/).

^b^ Please note that some markers may have additional microvariants.

^c^ Reference data for HuH-7, cells was obtained from Kasai et al. (2018).

### 3.4 Combined karyotype and M-FISH analysis

**FIGURE 3:**
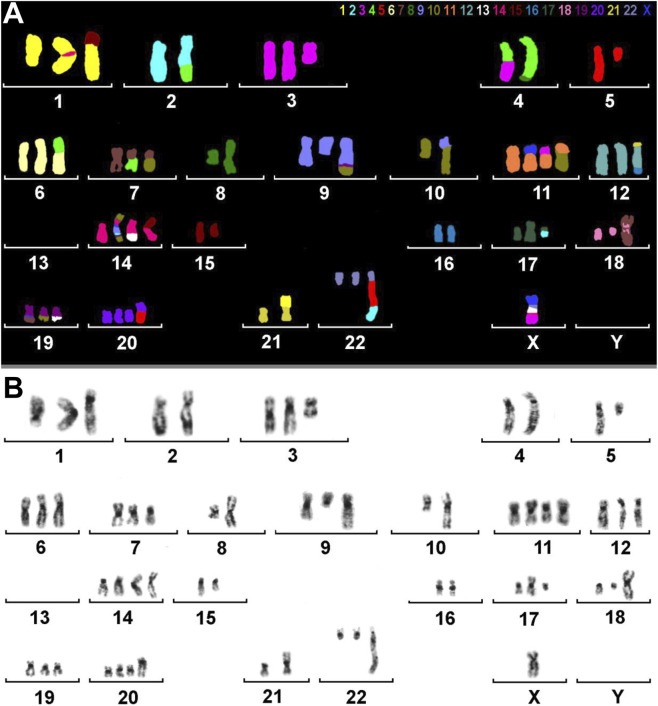
M-FISH and inverted DAPI-banding of HuH-7. **(A)** M-FISH result of a metaphase with colors assigned to each chromosome. Most chromosomes are derivatives consisting of parts from two or more original human chromosomes. The color key for each chromosome is provided at the top of the image. It is important to note that there is no Y chromosome present. **(B)** The same metaphase is displayed with inverted DAPI banding. The karyogram reveals chromosomal rearrangements and numerical abnormalities in HuH-7 cells.

A molecular cytogenetic analysis on the HuH-7 cell line was conducted to obtain a comprehensive understanding of its chromosomal composition. Utilizing inverted DAPI-banding in conjunction with M-FISH allowed for each chromosome to be visualized in a distinct color, adding in the identification and mapping of intrachromosomal genetic alterations and/or aneuploidies within the cells. Our analysis indicated that HuH-7 cells have an almost triploid karyotype (59<3n>), showcasing both numerical and highly complex structural aberrations in some parts (Figure 3).

**FIGURE 4:**
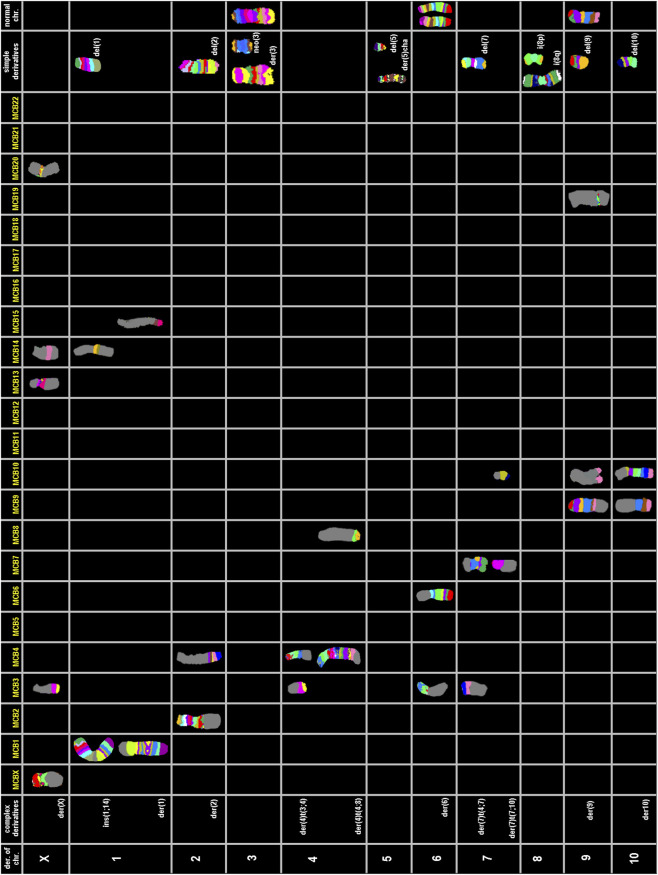
Multicolor banding (MCB) of HuH-7 chromosomes. Representative examples of high-resolution multicolor banding profiles of HuH-7 chromosomes are shown for derivatives of chromosomes X, 1 to 10.

**FIGURE 5:**
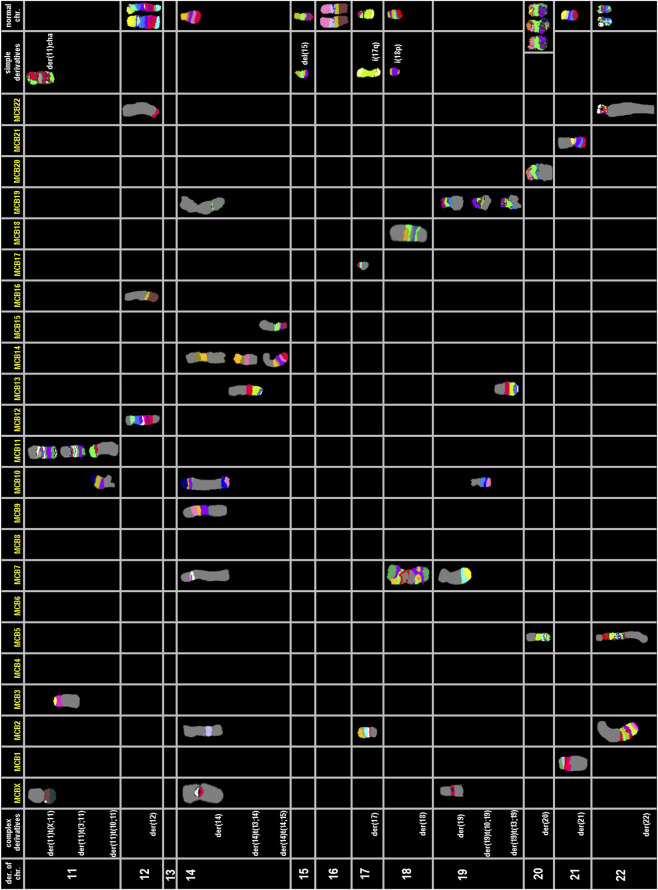
Multicolor banding (MCB) of HuH-7 chromosomes. Representative examples of high-resolution multicolor banding profiles of HuH-7 chromosomes are shown for derivatives of chromosomes 11 to 22.

To clarify the complex chromosomal rearrangements in HuH-7 cells, we performed a high-resolution, multicolor banding (MCB) analysis. This technique enables DNA-specific color banding along one chromosome at a time (Figures 4, 5). Notably, the Y-chromosome is absent, with only one derivative X chromosome present, although parts of X-chromosome(s) are also found on derivatives of chromosomes 11, 14, and 19. Chromosome 1 shows multiple rearrangements, including a deletion and an insertion involving chromosome 14, as well as a large derivative containing chromosome 15. Chromosome 2 displays deletions and a translocation to chromosome 4, leading to the complete loss of one copy. Several other chromosomes (3, 4, 5, 7, 9, 10, 11, 14 and 19) exhibit complex derivative forms, with merged, inverted, or inserted segments from multiple sources. For example, chromosome 3 has a derivative that incorporates multiple rearrangements and a neochromosome. Isochromosomes are observed in chromosomes 8, and 18. Chromosome 13 is absent, with some fragments replaced by translocated segments. Chromosome 14 shows significant rearrangements, while the remaining derivatives contain interspersed chromosome arms. Additional abnormalities include large deletions, missing chromosomes, and recurring translocations, such as with chromosomes 15, 16, 21, and 22. Moreover, several aberrations are so complex that they could not be fully resolved by FISH or aCGH (see below). Therefore, one derivative each of chromosome 5 and 11 were reported as having been going through ‘cha’ = chromoanasynthesis, which is a special form of chromothripsis.

Overall, the karyotype (also detailed by chromosome in Table 3) can be summarized as: 56<3n>,der(X) (Xpter- > Xq12::20p13->20p13::13q11.2->13q12::14q23->14q24.3::3q26->3qter),-X,-X,del (1) (q12),ins (1; 14) (q12; p1?2q11.2),der (1) (15qter->15q26.1::1q12->1q24::1q24->1q12::1q24->1qter),del (2) (p21),der (2)t (2; 4) (q14.1; q31),-2,3,der (3) (qter- > q28::p12- > q11.2::q28- > q29::q11.2- > qter),neo (3) (pter- > p21.3::p21.3- > pter),der (4) (3pter->3p21.3::4p16->4q12),der (4)t (4; 8) (q33; p23.1),-4,der (5)cha (q12->?qter),del (5) (q13),-5,6,6,der (6)t (4; 6) (p12; p11.2),del (7) (q22),der (7)t (4; 7) (q31; p11.2),der (7) (7p12->7q11.2::10p13->10pter),i (8) (p10),i (8) (q10),-8,9,del (9) (q12),der (9) (9pter->9q34.3::19q13->19q13::10q25.3->10qter),-9,del (10) (q23),der (10)t (9; 10) (q31; p11.2),-10,der (11)cha (pter- > q1?2),der (11)t (X; 11) (q26; p11.2),der (11)t (3; 11) (q26; p11.11),+der (11)t (10; 11) (q11.2; p14),12,12,der (12) (21qter->21q22.2 :: 12p11.2->12q23 ::16q11.2-> 16qter), -13, -13,-13,14,der (14) (10pter->10p11.2::7q11.21->7q11.21::14q11.2->14p12::Xp11.21- > Xp11.21::9q12->9q21::9q33->9q34::2p16->2p16::19p12->19p12::10q25.1->10qter),der (14)t (13; 14) (q11.2; q23),+der (14)t (14; 15) (p13; q11.2),del (15) (q22),-15,-16,17,i (17) (q10),der (17)t (2; 17) (p21; q12),18,i (18) (p10),der (18) (7qter->7q11.2::18q12->18p11.1::7q11.2->7q11.2::18q21->18q12::7q11.2->7qter),der (19) (19pter->19q13.4::Xq11.2- > Xq12::7p11.2->7pter),der (19)t (10; 19) (q24; q13.4),der (19)t (13; 19) (q11.2; q13.4),+der (20)t (5; 20) (q13; q12),der (21)t (1; 21) (p33; p13),-21,der (22) (22pter->22q11.2::5p13.2->5q35::2q14.1->2qter)[cp∼500].

**TABLE 3:** Summary of M−FISH and MCB analysis for HuH-7 cells.

Chromosome	Alterations
X	der(X) (Xpter- > Xq12::20p13->20p13::13q11.2->13q12::14q23->14q24.3::3q26->3qter)
1	del (1) (q12),ins (1; 14) (q12; p1?2q11.2),der (1) (15qter->15q26.1::1q12->1q24::1q24->1q12::1q24->1qter)
2	del (2) (p21),der (2)t (2; 4) (q14.1; q31)
3	3,der (3) (qter- > q28::p12- > q11.2::q28- > q29::q11.2- > qter),neo (3) (pter- > p21.3::p21.3- > pter)
4	der (4) (3pter->3p21.3::4p16->4q12),der (4)t (4; 8) (q33; p23.1
5	der (5)cha (q12->?qter),del (5) (q13)
6	6,6,der (6)t (4; 6) (p12; p11.2)
7	del (7) (q22),der (7)t (4; 7) (q31; p11.2),der (7) (7p12->7q11.2::10p13->10pter)
8	i (8) (p10),i (8) (q10)
9	9,del (9) (q12),der (9) (9pter->9q34.3::19q13->19q13::10q25.3->10qter)
10	del (10) (q23),der (10)t (9; 10) (q31; p11.2)
11	der (11)cha (pter- > q1?2),der (11)t (X; 11) (q26; p11.2),der (11)t (3; 11) (q26; p11.11), der (11)t (10; 11) (q11.2; p14)
12	12,12,der (12) (21qter->21q22.2::12p11.2->12q23::16q11.2->16qter)
13	13 n.a
14	14,der (14) (10pter->10p11.2::7q11.21->7q11.21::14q11.2->14p12::Xp11.21- > Xp11.21::9q12->9q21::9q33->9q34::2p16->2p16::19p12->19p12::10q25.1->10qter),der (14)t (13; 14) (q11.2; q23),der (14)t (14; 15) (p13; q11.2)
15	15,del (15) (q22)
16	16,16
17	17,i (17) (q10),der (17)t (2; 17) (p21; q12)
18	18,i (18) (p10),der (18) (7qter->7q11.2::18q12->18p11.1::7q11.2->7q11.2::18q21->18q12::7q11.2->7qter)
19	der (19) (19pter->19q13.4::Xq11.2- > Xq12::7p11.2->7pter),der (19)t (10; 19) (q24; q13.4),der (19)t (13; 19) (q11.2; q13.4)
20	20,20,20,der (20)t (5; 20) (q13; q12)
21	21,der (21)t (1; 21) (p33; p13)
22	22,22,der (22) (22pter->22q11.2::5p13.2->5q35::2q14.1->2qter)

In summary, HuH-7, cells exhibit pronounced genomic instability, as evidenced the numerous gains, losses, and complex translocations. This highly rearranged genome is a common feature of many tumor-derived cell lines, reflecting their aggressive and rapidly dividing nature. The karyotype provided is a “composite karyotype’ according to ISCN, 2024 (Hastings et al., 2024) from approximately ∼500 cells analyzed. Many single cell aberrations observed were not included in this analysis.

### 3.5 Array comparative genomic hybridization

**FIGURE 6:**
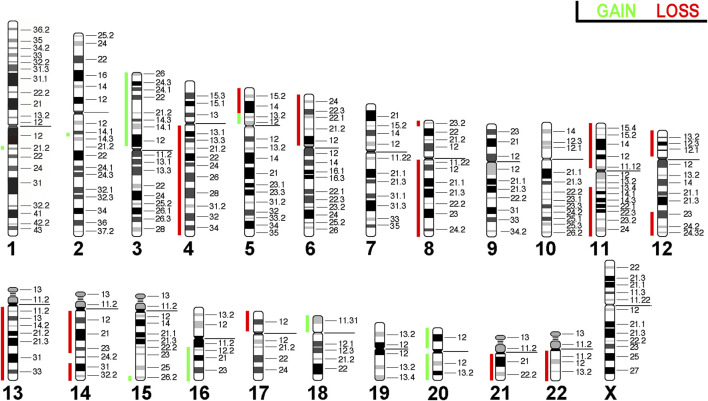
Array comparative genomic hybridization for the HuH-7 cell line. Copy number alterations are represented using a color code with red shades indicating losses and while green indicating gains.

Array comparative genomic hybridization (aCGH) was conducted on HuH-7 cells to visually map chromosomal gains and losses throughout the genome. This revealed the unique and complex genomic landscape of HuH-7 cells. Through aCGH analysis, we identified gains on the short arms of chromosomes 3, 5, 18, and 20, as well as on the long arms of chromosomes 15, 16, and 20. Conversely, losses were observed on the short arms of chromosomes 5, 6, 8, 11, 12, and 17, and on the long arms of chromosomes 4, 8, 11, 12, 13, 14, 21, and 22 (Figure 6). Overall, the results obtained here were consistent with the copy number alterations seen in molecular cytogenetic analyses. However, the complexity revealed in the molecular cytogenetic analyses was not fully captured in the aCGH profile.

As shown in Table 4, 15 out of 19 major copy number variations (CNVs) (approximately 80%) observed in HuH-7 are also found in human hepatocellular carcinoma, indicating that this cell line is a suitable model for advanced hepatocellular carcinoma.

**TABLE 4:** Losses and gains of chromosomal regions larger than one cytoband in HuH-7 cells compared with copy number alterations in hepatocellular carcinoma.

Cytogenetic span	Copy number variation in HuH-7	Copy number variation in hepatocellular carcinoma (Guan et al., 2000)	Concordance
3pter-3p12	Gain	Loss and gain	(+)
4q12-4qter	Loss	loss	+
5pter-5p14	Loss	Gain	−
5p13.2-5p12	Gain	Gain	+
6pter-6p11.2	Loss	Gain	−
8q11.21-8qter	Loss	Gain	−
11pter-11p11.11	Loss	Loss	+
11q13.4–11qter	Loss	Loss and gain	(+)
12pter-12p11.2	Loss	Loss and gain	(+)
12q23-12qter	Loss	Gain	−
13pter-13qter	Loss	Loss and gain	(+)
14q11.2–14q24.1	Loss	Loss	+
14q31-14qter	Loss	Loss	+
16q12.1–16qter	Gain	Loss	−
17pter-17p11.2	Loss	Loss	+
18pter-18p11.21	Gain	Loss and gain	(+)
20pter-20qter	Gain	Gain	+
21pter-21qter	Loss	Loss	+
22pter-22qter	Loss	Loss	+

### 3.6 Next-generation mRNA bulk sequencing

To gain an unbiased overview of the transcriptomic landscape of HuH-7 cells and accurately assess their mRNA expression capacity, we conducted next-generation mRNA bulk sequencing. This approach involves isolating and sequencing the entire mRNA pool, providing a comprehensive snapshot of gene expression in the cells when cultured in a standard medium. Capturing both abundant and lowly expressed transcripts provides valuable insight into the molecular pathways, potential biomarkers, and regulatory networks underlying cell function and disease mechanisms. This information is particularly important for understanding the susceptibility of HuH-7 cells to the hepatitis C virus (HCV).

Our analysis revealed several groups of highly expressed genes in HuH-7 cells. Firstly, there was strong representation of mitochondrial transcripts, including subunits of the electron transport chain (e.g., *MT-CO1*, *MT-CO2*, *MT-CO3*, *MT-ND1*, *MT-ND2*, *MT-ATP6*, and *MT-ATP8*), reflecting the active metabolic state of these cells. Secondly, numerous ribosomal proteins (e.g., *RPL41*, *RPS12*, *RPL37*, *RPS17*, and *RPS27*) and translation factors, such as eukaryotic translation elongation factor 1 alpha 1 (*EEF1A1)* and eukaryotic translation elongation factor 2 (*EEF2*), exhibited robust expression, suggesting significant protein synthesis activity (Supplementary Table S1). A further notable category comprised essential housekeeping and cytoskeletal genes, including glyceraldehyde-3-phosphate dehydrogenase (*GAPDH*), actin beta (*ACTB*), tubulin alpha 1b (*TUBA1B*), vimentin (*VIM*), the biliary/hepatic progenitor cell marker keratin 19 (*KRT19*), and keratins 18 (*KRT18*), and 8 (*KRT8*). These genes are indicative of fundamental cellular processes. Similarly, HuH-7 cells exhibited high mRNA expression of the transferrin receptor 1 (*TFRC*), which is a hepatitis C virus entry factor (Martin and Uprichard, 2013). HuH-7 cells also express scavenger receptor class B member 1 (*SCARB1*), which has also been reported to act as a hepatitis C virus receptor that and facilitates its entry into cells (Arandhara et al., 2023). Other genes that are thought to act as hepatitis C virus entry factors such as the CD81 molecule, occludin (*OCLN*), and claudin-1 (*CLDN1*), as well as the cofactor epidermal growth factor receptor (*EGFR*) (Carriquí-Madronal et al., 2023), are expressed at high levels (*CD81*, *CLDN1*, and *EGFR*) or median levels (*OCLN*) in HuH-7 cells (Supplementary Table S1).

Furthermore, a high expression of α1-antitrypsin, encoded by the serpin family A member 1 (*SERPINA1*) gene, was observed. This confirms previous data showing that HuH-7 cells can secrete large quantities of this protein (Ke et al., 2022). Considerable amounts of mRNA encoding the hepatocyte differentiation marker nuclear factor 4α (*HNF4A*) were also found. HNF4A regulates many genes involved in lipid and carbohydrate metabolism, including those that control very low-density lipoprotein (VLDL) secretion and gluconeogenesis (Huck et al., 2021). Ferritin heavy chain 1 (*FTH1*) and ferritin light chain (*FTL*) were expressed at high mRNA levels. These proteins form a complex that stores iron in a non-toxic form, and are predominantly expressed in the liver (Gao et al., 2022). Lastly, markers predominantly expressed in hepatocytes were found among the most highly expressed transcripts, which is consistent with the hepatocellular carcinoma origin of HuH-7 cells. Notably, HuH-7 cells exhibited high levels of expression of both albumin (*ALB*) and α-fetoprotein (*AFP*) (Table 5). However, cytochrome P450 (*CYP*) genes, such as *CYP3A4*, which are mainly responsible for the phase I metabolism of xenobiotics in hepatocytes, were found to be expressed at low levels. This confirms a previous report demonstrating that HuH-7 cells express negligible amounts of CYP450 enzymes (Bulutoglu et al., 2019). Under the chosen condition, HuH-7 cells also express mRNA of several transporters relevant for drug metabolism at low level, including organic anion transporter polypeptides (OATP1A2/*SLCO1A2*, OATP1B1/*SLCO1B1*, OATPB3/*SLC01B3*), organic cation transporters (OCT1/*SLC22A1*), multidrug-resistance-associated proteins (MRP2/*ABCC2*, MRP3/*ABCC3*, MRP4/*ABCC4*), bile salt export pumps (*ABCB11*), and many others (Supplementary Table S1).

**TABLE 5:** Selected gene expression in HuH-7 cells that underpins their hepatocytic origin^a^.

*Gene*	Gene description	Gene Id	Transcript Id	TPM^b^
*ABCC3*	ATP binding cassette subfamily C member 3	ENSG00000108846	ENST00000285238.13 ENST00000427699.5 ENST00000515707.1	5.411044 1.108026 0.275668
*ABCD3*	ATP binding cassette subfamily D member 3	ENSG00000117528	ENST00000370214.9 ENST00000315713.5	109.991235 17.535516
*ACADM*	Acyl-CoA dehydrogenase medium chain	ENSG00000117054	ENST00000370841.9 ENST00000420607.6 ENST00000680964.1 ENST00000680805.1 ENST00000679687.1	57.964581 38.1719 1.343853 0.984129 0.639576
*ACAT2*	Acetyl-CoA acetyltransferase 2	ENSG00000120437	ENST00000367048.5	490.01092
*ACLY*	ATP citrate lyase	ENSG00000131473	ENST00000352035.7 ENST00000353196.5 ENST00000590151.5 ENST00000393896.6	145.333931 86.238545 74.594288 57.214373
*ACSS2*	Acyl-CoA synthetase short chain family member 2	ENSG00000131069	ENST00000360596.7 ENST00000253382.5	52.484305 0.241686
*AFP*	Alpha fetoprotein	ENSG00000081051	ENST00000395792.7 ENST00000226359.2	1068.981533 784.704493
*AHR*	Aryl hydrocarbon receptor	ENSG00000106546	ENST00000242057.9 ENST00000642825.1	46.985221 1.09226
*A1CF*	APOBEC1 complementation factor	ENSG00000148584	ENST00000374001.6 ENST00000373993.6 ENST00000373997.8 ENST00000395489.7 ENST00000373995.7	26.589698 14.764128 3.52316 1.437412 0.155239
*AKR1C1*	Aldo-keto reductase family 1 member C1	ENSG00000187134	ENST00000380859.1 ENST00000380872.9	4.137038 2.082184
*ALB*	Albumin	ENSG00000163631	ENST00000509063.5 ENST00000415165.6 ENST00000295897.9 ENST00000401494.7 ENST00000503124.5	5026.854128 2410.505634 1856.352717 11.497756 3.287102
*ALDH6A1*	Aldehyde dehydrogenase 6 family member A1	ENSG00000119711	ENST00000553458.6 ENST00000350259.8 ENST00000555126.1	9.989024 8.117909 0.495415
*AMBP*	Alpha-1-microglobulin/bikunin precursor	ENSG00000106927	ENST00000265132.8	1004.193092
*ANG*	Angiogenin	ENSG00000214274	ENST00000336811.10 ENST00000397990.5	19.245275 5.390933
*ANXA13*	Annexin A13	ENSG00000104537	ENST00000419625.6 ENST00000262219.10	2.017452 1.684361
*APOA1*	Apolipoprotein A1	ENSG00000118137	ENST00000236850.5 ENST00000375323.5 ENST00000359492.6 ENST00000375320.5 ENST00000375329.6	479.580739 41.49574 19.352689 6.12056 5.409424
*APOA2*	Apolipoprotein A2	ENSG00000158874	ENST00000367990.7 ENST00000468465.5 ENST00000463812.1 ENST00000464492.5	851.641601 23.30225 19.396845 2.461307
*APOA5*	Apolipoprotein A5	ENSG00000110243	ENST00000227665.9	0.551303
*APOB*	Apolipoprotein B	ENSG00000084674	ENST00000233242.5 ENST00000399256.4	418.665918 0.770799
*APOC3*	Apolipoprotein C3	ENSG00000110245	ENST00000227667.8	101.38132
*APOH*	Apolipoprotein H	ENSG00000091583	ENST00000205948.11	97.622672
*APOM*	Apolipoprotein M	ENSG00000204444	ENST00000375916.4 ENST00000375918.6	40.482114 1.551169
*AQP3*	Aquaporin 3 (Gill blood group)	ENSG00000165272	ENST00000297991.6	28.9842
*ARG1*	Arginase 1	ENSG00000118520	ENST00000368087.8 ENST00000356962.2 ENST00000673427.1	27.344412 0.562976 0.229806
*ASGR1*	Asialoglycoprotein receptor 1	ENSG00000141505	ENST00000269299.8 ENST00000619926.4 ENST00000572879.5 ENST00000574388.5	25.499817 4.861638 1.92106 1.385759
*ASL*	Argininosuccinate lyase	ENSG00000126522	ENST00000395332.8 ENST00000673518.1 ENST00000380839.9 ENST00000304874.14 ENST00000395331.4	39.212551 21.491007 6.070932 4.366237 0.314354
*ASS1*	Argininosuccinate synthase 1	ENSG00000130707	ENST00000372394.5 ENST00000372393.7 ENST00000352480.10	400.899918 22.238217 0.793815
*ATP7B*	ATPase copper transporting beta	ENSG00000123191	ENST00000242839.10 ENST00000673772.1 ENST00000448424.7	6.866589 0.866004 0.524998
*BNIP3*	BCL2 interacting protein 3	ENSG00000176171	ENST00000368636.9 ENST00000633835.2 ENST00000540159.4	308.766968 10.429135 6.933424
*C1ORF53*	Chromosome 1 open reading frame 53	ENSG00000203724	ENST00000367393.8	5.135328
*C4B*	Complement C4B	ENSG00000224389	ENST00000435363.7	4.643549
*CDH1*	Cadherin 1	ENSG00000039068	ENST00000261769.10 ENST00000422392.6	53.193162 0.905931
*CEBPA*	CCAAT enhancer binding protein alpha	ENSG00000245848	ENST00000498907.3	283.638502
*CP*	Ceruloplasmin	ENSG00000047457	ENST00000264613.11	11.108003
*CPS1*	Carbamoyl-phosphate synthase 1	ENSG00000021826	ENST00000233072.10 ENST00000673510.1	0.608039 0.467479
*CRP*	C-reactive protein	ENSG00000132693	ND	0
*CTNNB1*	Catenin beta 1	ENSG00000168036	ENST00000396185.8 ENST00000646725.1 ENST00000645982.1 ENST00000643031.1 ENST00000647390.1 ENST00000643992.1 ENST00000642886.1 ENST00000643297.1 ENST00000647264.1 ENST00000642426.1 ENST00000645276.1 ENST00000644873.1 ENST00000647413.2 ENST00000644138.1 ENST00000645320.1 ENST00000431914.6 ENST00000644867.1 ENST00000646174.1 ENST00000644524.1 ENST00000645493.1	74.324211 63.255902 15.640361 13.958452 11.670908 4.461679 3.900712 3.194732 2.363529 2.238538 2.12679 1.31504 0.899867 0.695768 0.531144 0.420068 0.271178 0.227283 0.154312 0.139239
*CYP1A1*	Cytochrome P450 family 1 subfamily A member 1	ENSG00000140465	ENST00000379727.8 ENST00000395048.6	7.357747 1.700431
*CYP1A2*	Cytochrome P450 family 1 subfamily A member 2	ENSG00000140505	ENST00000343932.5	0.022769
*CYP2A6*	Cytochrome P450 family 2 subfamily A member 6	ENSG00000255974	ENST00000301141.10	0.083751
*CYP2A7*	Cytochrome P450 family 2 subfamily A member 7	ENSG00000198077	ENST00000301146.9	0.08567
*CYP2B6*	Cytochrome P450 family 2 subfamily B member 6	ENSG00000197408	ENST00000324071.10 ENST00000593831.1	4.007195 2.121862
*CYP2C8*	Cytochrome P450 family 2 subfamily C member 8	ENSG00000138115	ENST00000535898.5	0.248138
*CYP2C9*	Cytochrome P450 family 2 subfamily C member 9	ENSG00000138109	ENST00000260682.8	0.366568
*CYP2C19*	Cytochrome P450 family 2 subfamily C member 19	ENSG00000165841	ENST00000371321.9	0.136255
*CYP2D6*	Cytochrome P450 family 2 subfamily D member 6	ENSG00000100197	ENST00000359033.4	0.676141
*CYP2E1*	Cytochrome P450 family 2 subfamily E member 1	ENSG00000130649	ND	0
*CYP3A4*	Cytochrome P450 family 3 subfamily A member 4	ENSG00000160868	ND	0
*CYP3A7*	Cytochrome P450 family 3 subfamily A member 7	ENSG00000160870	ND	0
*CYP7A1*	Cytochrome P450 family 7 subfamily A member 1	ENSG00000167910	ND	0
*DEFB1*	Defensin beta 1	ENSG00000164825	ENST00000297439.4	15.657857
*ENTPD5*	Ectonucleoside triphosphate diphosphohydrolase 5 (inactive)	ENSG00000187097	ENST00000334696.11 ENST00000557325.5 ENST00000556242.5	52.760183 3.30735 0.951792
*EPB41L4B*	Erythrocyte membrane protein band 4.1 like 4B	ENSG00000095203	ENST00000374557.4 ENST00000374566.8	12.995235 0.130356
*EPPK1*	Epiplakin 1	ENSG00000261150	ENST00000615648.2	5.282482
*FABP1*	Fatty acid binding protein 1	ENSG00000163586	ENST00000295834.8 ENST00000393750.3	1022.282132 1.866548
*FGA*	Fibrinogen alpha chain	ENSG00000171560	ENST00000403106.8 ENST00000651975.2	804.238787 67.580784
*FGB*	Fibrinogen beta chain	ENSG00000171564	ENST00000509493.1 ENST00000302068.9	723.333107 224.64291
*FGG*	Fibrinogen gamma chain	ENSG00000164687	ENST00000404648.7 ENST00000407946.5 ENST00000405164.5 ENST00000336098.8	373.801269 59.853934 40.068397 8.820809
*FGL1*	Fibrinogen like 1	ENSG00000104760	ENST00000427924.5 ENST00000518650.5 ENST00000381841.4 ENST00000398056.6	27.820338 8.833352 4.253756 0.806466
*FGFR4*	Fibroblast growth factor receptor 4	ENSG00000160867	ENST00000292408.9 ENST00000502906.5 ENST00000393648.6 ENST00000393637.5	157.688635 69.280419 20.030395 12.297557
*FN1*	Fibronectin 1	ENSG00000115414	ENST00000354785.11 ENST00000443816.5 ENST00000446046.5 ENST00000323926.10 ENST00000432072.6 ENST00000426059.1	445.829638 222.634594 132.240541 48.233483 31.046218 1.288296
*FOXA1*	Forkhead box A1	ENSG00000129514	ENST00000250448.5	33.614447
*FOXA2*	Forkhead box A2	ENSG00000125798	ENST00000377115.4 ENST00000419308.7	61.98258 26.298633
*FOXA3*	Forkhead box A3	ENSG00000170608	ENST00000302177.3	29.942726
*FST*	Follistatin	ENSG00000134363	ENST00000256759.8 ENST00000396947.7	16.315592 3.179729
*FTH1*	Ferritin heavy chain 1	ENSG00000167996	ENST00000620041.5 ENST00000273550.12 ENST00000526640.5 ENST00000529631.5 ENST00000532601.1 ENST00000529191.5	3617.934969 2430.160321 468.800171 7.988079 5.402953 0.753325
*FTL*	Ferritin light chain	ENSG00000087086	ENST00000331825.11	4436.265555
*G0S2*	G0/G1 switch 2	ENSG00000123689	ENST00000367029.5	0.85612
*GC*	GC vitamin D binding protein	ENSG00000145321	ENST00000273951.13	1.911797
*GCK*	Glucokinase	ENSG00000106633	ND	0
*GHR*	Growth hormone receptor	ENSG00000112964	ENST00000230882.9 ENST00000537449.5	1.556932 1.50656
*GJB2*	Gap junction protein beta 2	ENSG00000165474	ENST00000382848.5	1.780546
*GLS2*	Glutaminase 2	ENSG00000135423	ENST00000623608.3 ENST00000311966.9	0.336513 0.221366
*GPAT4*	Glycerol-3-phosphate acyltransferase 4	ENSG00000158669	ENST00000396987.7	33.732265
*GPC3*	Glypican 3	ENSG00000147257	ENST00000370818.8 ENST00000631057.2 ENST00000689310.1 ENST00000394299.7	567.58236 29.821612 19.307112 6.864262
*GLUL*	Glutamate-ammonia ligase	ENSG00000135821	ENST00000339526.8 ENST00000331872.11 ENST00000417584.6 ENST00000311223.9	174.690605 14.633245 6.081557 0.359024
*GOLT1A*	Golgi transport 1A	ENSG00000174567	ENST00000308302.4	17.050775
*GRB14*	Growth factor receptor bound protein 14	ENSG00000115290	ENST00000263915.8 ENST00000696453.2	7.605801 5.64976
*GRP*	Gastrin releasing peptide	ENSG00000134443	ND	0
*GSTA2*	Glutathione S-transferase alpha 2	ENSG00000244067	ENST00000493422.3	0.320086
*HAL*	Histidine ammonia-lyase	ENSG00000084110	ENST00000261208.8 ENST00000538703.5 ENST00000541929.5	12.438387 10.538754 1.360067
*HAMP*	Hepcidin antimicrobial peptide	ENSG00000105697	ENST00000222304.5	0.559711
*HMGCS1*	3-hydroxy-3-methylglutaryl-CoA synthase 1	ENSG00000112972	ENST00000433297.2 ENST00000325110.11	754.36224 35.582875
*HNF4A*	Hepatocyte nuclear factor 4 alpha	ENSG00000101076	ENST00000316673.9 ENST00000316099.10 ENST00000415691.2 ENST00000443598.6	48.552653 40.712645 10.090812 2.145697
*HP*	Haptoglobin	ENSG00000257017	ENST00000355906.10 ENST00000398131.6	6.426395 2.581071
*HPR*	Haptoglobin-related protein	ENSG00000261701	ENST00000540303.7	3.849199
*KIF13B*	Kinesin family member 13B	ENSG00000197892	ENST00000524189.6 ENST00000521515.1	14.885443 1.387584
*KRT8*	Keratin 8	ENSG00000170421	ENST00000692008.1 ENST00000552150.5 ENST00000552551.5 ENST00000619952.2 ENST00000293308.11	3054.08644199999 26.824888 25.416126 2.679175 2.174108
*KRT18*	Keratin 18	ENSG00000111057	ENST00000388835.4 ENST00000388837.6 ENST00000550600.5	2392.850981 38.581545 4.101943
*KRT19*	Keratin 19	ENSG00000171345	ENST00000361566.7	23.284769
*LEPR*	Leptin receptor	ENSG00000116678	ENST00000371060.7 ENST00000616738.4 ENST00000371059.7 ENST00000371058.1 ENST00000349533.11	5.233358 3.527942 2.285827 0.812115 0.22691
*LIPC*	Lipase C, hepatic type	ENSG00000166035	ENST00000588188.7 ENST00000356113.10 ENST00000299022.10 ENST00000414170.7	79.911997 28.699433 11.889459 0.852596
*LRP5*	LDL receptor related protein 5 [	ENSG00000162337	ENST00000294304.12	140.209953
*MCC*	MCC regulator of WNT signaling pathway	ENSG00000171444	ENST00000302475.9 ENST00000514701.5	11.382496 0.182403
*MSMO1*	Methylsterol monooxygenase 1	ENSG00000052802	ENST00000261507.11 ENST00000393766.6 ENST00000504317.1	287.11002 29.949633 2.293034
*NOS2*	Nitric oxide synthase 2	ENSG00000007171	ENST00000697339.1	0.074539
*OCIAD1*	OCIA domain containing 1	ENSG00000109180	ENST00000513391.2 ENST00000396448.6 ENST00000381473.7 ENST00000264312.12 ENST00000508293.5 ENST00000444354.6	33.816391 8.933271 3.947959 1.77301 0.29131 0.190061
*ORM1*	Orosomucoid 1	ENSG00000229314	ENST00000259396.9	173.756453
*OTC*	Ornithine transcarbamylase	ENSG00000036473	ENST00000039007.5	0.176958
*PAH*	Phenylalanine hydroxylase	ENSG00000171759	ENST00000307000.7 ENST00000553106.6	52.748713 16.981983
*PCK1*	Phosphoenolpyruvate carboxykinase 1	ENSG00000124253	ENST00000319441.6	0.016257
*PHLDA1*	Pleckstrin homology like domain family A member 1	ENSG00000139289	ENST00000602540.5 ENST00000266671.10	14.406729 0.606953
*PLIN1*	Perilipin 1	ENSG00000166819	ENST00000430628.2	0.197608
*PLSCR1*	Phospholipid scramblase 1	ENSG00000188313	ENST00000487389.5 ENST00000342435.9 ENST00000448787.6	9.816466 7.509352 1.71921
*PON3*	Paraoxonase 3	ENSG00000105852	ENST00000265627.10 ENST00000451904.5	5.899761 0.263607
*PRRG4*	Proline rich and Gla domain 4	ENSG00000135378	ENST00000257836.4	0.728434
*RHOB*	Ras homolog family member B	ENSG00000143878	ENST00000272233.6	91.588906
*RND3*	Rho family GTPase 3	ENSG00000115963	ENST00000263895.9 ENST00000375734.6	28.855417 7.529461
*RPP25L*	Ribonuclease p/mrp subunit p25 like	ENSG00000164967	ENST00000378959.9 ENST00000297613.4	12.579198 3.391974
*SAA4*	Serum amyloid a4, constitutive	ENSG00000148965	ENST00000278222.7	1.164446
*SAT2*	Spermidine/spermine N1-acetyltransferase family member 2	ENSG00000141504	ENST00000269298.10 ENST00000573566.1	50.874831 22.663125
*SCARB1*	Scavenger receptor class B member 1	ENSG00000073060	ENST00000261693.11 ENST00000415380.6 ENST00000546215.5 ENST00000680556.1 ENST00000680596.1 ENST00000544327.1	91.542205 68.506383 59.199431 26.369478 6.578246 4.461932
*SCD*	Stearoyl-CoA desaturase	ENSG00000099194	ENST00000370355.3	1147.411199
*SDC2*	Syndecan 2	ENSG00000169439	ENST00000302190.9 ENST00000522911.5 ENST00000518385.5	39.821114 24.342346 11.471846
*SERPINA1*	Serpin family A member 1 (α1-antitrypsin)	ENSG00000197249	ENST00000636712.1 ENST00000393087.9 ENST00000402629.1 ENST00000393088.8 ENST00000437397.5 ENST00000448921.5 ENST00000440909.5	2162.842686 19.373091 4.135652 1.256812 1.110108 0.346714 0.212755
*SERPINH1*	Serpin family H member 1	ENSG00000149257	ENST00000358171.8 ENST00000533603.5 ENST00000524558.5 ENST00000530284.5	239.676154 11.505526 1.122409 0.133934
*SFRP5*	Secreted frizzled related protein 5	ENSG00000120057	ENST00000266066.4	2.202643
*SLC2A2*	Solute carrier family 2 member 2	ENSG00000163581	ENST00000314251.8	0.582367
*SLC10A1*	Solute carrier family 10 member 1	ENSG00000100652	ND	0
*SPTBN1*	Spectrin beta, non-erythrocytic 1	ENSG00000115306	ENST00000356805.9 ENST00000333896.5	171.825791 81.986192
*SULT1A1*	Sulfotransferase family 1A member 1	ENSG00000196502	ENST00000314752.12 ENST00000569554.5	9.332073 0.184844
*TAT*	Tyrosine aminotransferase	ENSG00000198650	ENST00000355962.5	2.269519
*TF*	Transferrin	ENSG00000091513	ENST00000402696.9	195.691322
*TFR2*	Transferrin receptor 2	ENSG00000106327	ENST00000462107.1	7.812334
*TFRC*	Transferrin receptor	ENSG00000072274	ENST00000360110.9 ENST00000392396.7 ENST00000420415.5 ENST00000698290.1 ENST00000698285.1 ENST00000698291.1 ENST00000698295.1 ENST00000698280.1	468.574101 10.269163 2.467132 1.365116 1.115318 0.747771 0.57931 0.333346
*TKFC*	Triokinase and FMN cyclase	ENSG00000149476	ENST00000394900.8	27.045395
*TM4SF4*	Transmembrane 4 L six family member 4	ENSG00000169903	ENST00000305354.5	4.740282
*TM4SF5*	Transmembrane 4 L six family member 5	ENSG00000142484	ENST00000270560.4	52.256383
*TM7SF2*	Transmembrane 7 superfamily member 2	ENSG00000149809	ENST00000279263.14 ENST00000612081.1	118.745948 1.18996
*TMEM97*	Transmembrane protein 97	ENSG00000109084	ENST00000226230.8 ENST00000336687.6 ENST00000582113.1	327.625775 37.141432 1.365351
*TP53INP2*	Tumor protein p53 inducible nuclear protein 2	ENSG00000078804	ENST00000374810.8 ENST00000374809.6	23.691592 4.407257
*TTC36*	Tetratricopeptide repeat domain 36	ENSG00000172425	ENST00000302783.10	0.134539
*TTR*	Transthyretin	ENSG00000118271	ENST00000237014.8 ENST00000649620.1	333.165604 1.859561
*TUBA1B*	Tubulin alpha 1b	ENSG00000123416	ENST00000336023.9	3272.391142
*UCP2*	Uncoupling protein 2	ENSG00000175567	ENST00000663595.2 ENST00000536983.5	39.791085 1.472098
*UGT1A1*	UDP glucuronosyltransferase family 1 member A1	ENSG00000241635	ENST00000305208.10 ENST00000360418.4	1.281165 0.093469
*VDR*	Vitamin D receptor	ENSG00000111424	ENST00000549336.6	0.622198
*VIM*	Vimentin	ENSG00000026025	ENST00000224237.9 ENST00000339485.4	2006.517712 1.493837
*WT1*	WT1 transcription factor	ENSG00000184937	ND	0
*WTAP*	WT1 associated protein	ENSG00000146457	ENST00000337387.4 ENST00000621533.5 ENST00000614346.4 ENST00000631126.2	36.19295 24.856099 11.36496 2.759872
*ZHX2*	Zinc fingers and homeoboxes 2	ENSG00000178764	ENST00000314393.6	26.416903
*ACTB*	Actin beta	ENSG00000075624	ENST00000646664.1 ENST00000676397.1 ENST00000493945.6 ENST00000432588.6 ENST00000642480.2 ENST00000473257.3 ENST00000675515.1	3716.97217 33.690344 23.548124 6.407391 5.493343 2.582519 0.164191
*GAPDH*	Glyceraldehyde-3-phosphate dehydrogenase	ENSG00000111640	ENST00000229239.10 ENST00000396856.5 ENST00000396859.5 ENST00000619601.1 ENST00000396861.5 ENST00000396858.5	5285.775364 31.631692 31.531195 16.213084 15.90503 0.202033

^a^ Please note that, although the depicted genes are often described as hepatocyte-specific in various studies, many of these genes are also present in other liver cells. For example, the scavenger receptor class B member 1 (SCARB1), also referred to as SR-B1, is highly expressed in liver sinusoidal endothelial cells (Ganesan et al., 2016).

^b^ To compare the transcript levels of the listed genes, the expressions of actin beta (ACTB) and glyceraldehyde-3-phosphate dehydrogenase (GAPDH) are shown. The complete mRNA, expression profile of HuH-7 cells observed by NGS, can be found in Supplementary Table S1. Abbreviations used: ND, no transcripts of this gene were detected; TPM, transcripts per million.

### 3.7 Western blot analysis

**FIGURE 7:**
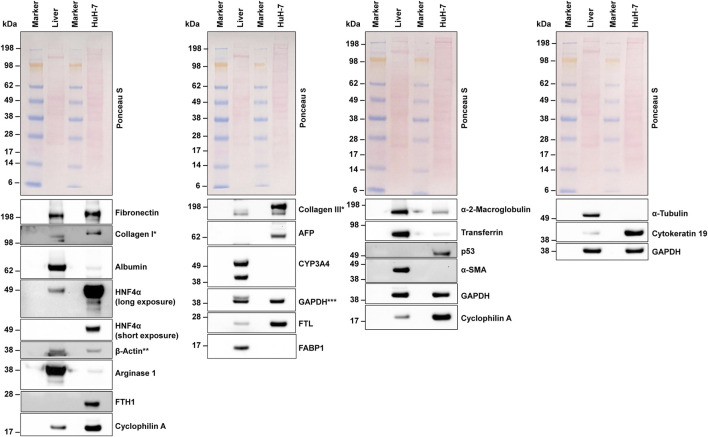
Western blot analysis. Protein extracts from HuH-7 cells and human liver tissue were analyzed using Western blot analysis. Equal protein loading was confirmed through Ponceau S staining and probing with antibodies for housekeeping proteins. A variety of proteins were detected, including important hepatic markers like albumin, hepatocyte nuclear factor 4 alpha (HNF4α), and α-fetoprotein (AFP). * In the extracts tested, collagens showed a multiple band pattern. A similar pattern was found in the murine hepatocyte cell line AML12 and murine hepatic stellate cell line GRX (Schröder et al., 2022). ** The lower signal for β-actin in the liver extracts resulted from former probing for Arginase 1. *** The upper signal for GAPDH in the liver extracts resulted from former probing with CYP3A4. Abbreviations: α-SMA, α-smooth muscle actin; CYP3A4, cytochrome P450 family 3 subfamily A member 4; FABP1, fatty acid binding protein 1; FTH1, Ferritin heavy chain 1; FTL, ferritin light chain; GAPDH, glyceraldehyde-3-phosphate dehydrogenase.

To ensure the accuracy of our next-generation mRNA sequencing results, we performed Western blot analyses on selected proteins of interest (Figure 7), using a protein extract from human liver tissue as a control. In most cases, the Western blot results were consistent with our NGS findings, confirming that many of the transcripts detected at the RNA level were indeed translated into proteins. Specifically, we observed strong expression of fibronectin, HNF4α, β-actin, FTH1, cyclophilin A, collagen type III, AFP, GAPDH, FTL, and (cyto)keratin 19. In line with our NGS data, we demonstrated that CYP3A4 expression was rather low. However, despite the presence of relatively high quantities of fatty acid binding protein 1 (*FABP1)* mRNA, the FABP1 protein was either undetectable or very low. Interestingly, HuH-7 cells also expressed the tumor marker p53, consistent with the mRNA data (Supplementary Table S1). Overall, the Western blot data predominantly confirmed the transcript-level findings from our NGS analysis, demonstrating that most of the identified mRNAs were translated into proteins. This strong alignment underscores the reliability of our NGS data.

### 3.8 Electron microscopic analysis

**FIGURE 8:**
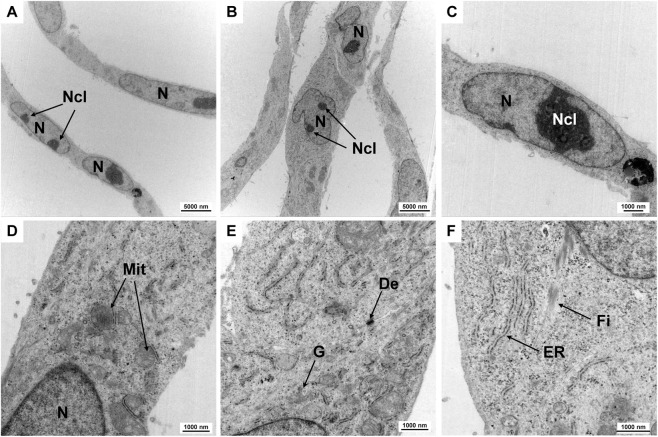
Ultrastructural examination using TEM. Transmission electron micrographs of the subcellular organelles in HuH-7 cells are shown, including mitochondria (Mit), endoplasmic reticulum (ER), Golgi apparatus (G), desmosome (De), and fibers. Some nuclei contain two nucleoli (Ncl). Characteristic fibers (Fi) are visible. Scale bars are 5,000 nm for images **(A,B)**, and 1,000 nm for images **(C–F)**. Magnifications are as follows: **(A,B)** ×2,784, **(C)** 7,750×, **(D,E)** 10,000×, and **(F)** 12,930×.

Electron microscopy was used to characterize the ultrastructure of HuH-7 cells (Figure 8). As expected for a hepatocyte-derived cell line, the cells exhibited numerous well-organized organelles associated with protein synthesis and detoxification. The large number of mitochondria reflects the high metabolic demand of these cells. Additionally, cells also exhibited prominently developed rough endoplasmic reticulum and a distinct Golgi apparatus. Similarly, the nuclei of the cells have a lot of euchromatin and pronounced, often two, nucleoli, which also indicate a high metabolic activity of the cells. Some cells also showed evidence of glycogen granules and lipid droplets in certain areas, both of which are characteristic features of hepatocytes involved in energy storage and lipid metabolism. Fibers typical of keratin bundles were also found inside the cells. Moreover, cell junctions were visible between neighboring cells, suggesting preserved epithelial characteristics. However, these junctions may be less extensive *in vitro* than in primary liver tissue. Overall, these ultrastructural traits corroborate the hepatocellular identity of HuH-7 cells and align with the known morphology of parenchymal liver cells.

### 3.9 Cytoskeleton of HuH-7 cells

**FIGURE 9:**
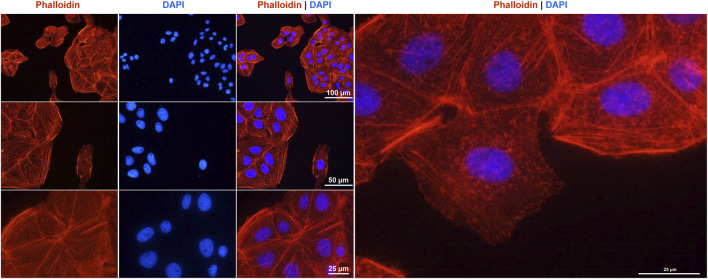
F-actin cytoskeleton staining in HuH-7 cells. The F- actin fibers in the cultured cells were labeled with a Rhodamine-Phalloidin probe (red) and the nuclei were counterstained with DAPI (blue). Images were captured using a Nikon Eclipse E80i fluorescence microscope at magnifications of ×200, ×400 and ×600 magnification. Scale bars (25 μm, 50 μm, and 100 µm) at different magnifications are shown.

Finally, we used Rhodamine-Phalloidin staining to visualize the organization of the actin cytoskeleton in HuH-7 cells, revealing their characteristic epithelial morphology (Figure 9). As expected for a hepatocyte-derived cell line, the cells exhibited a prominent cortical actin belt. This helps maintain a polygonal cell shape and facilitates strong cell-to-cell contacts in order to form monolayers. Additionally, F-actin stress fibers were visible throughout the cytoplasm, reflecting the dynamic and contractile properties of the cellular actin network. Some cells also displayed peripheral ruffling, indicating ongoing membrane remodeling activities that support cell motility and adhesion. Overall, this cytoskeletal arrangement is consistent with the hepatocellular origin of HuH-7 cells, emphasizing their capacity to maintain robust structural integrity in culture.

### 3.10 Markers of hepatic stellate cells, Kupffer cells and liver sinusoidal endothelial cells

In our analyses, markers specific to hepatic stellate cells (HSCs) (Supplementary Table S2), Kupffer cells (Supplementary Table S3), and liver sinusoidal endothelial cells (LSECs) (Supplementary Table S4) were either absent or present only minimally in HuH-7 cells. This finding supports the conclusion that HuH-7 cells predominantly exhibit a hepatocellular phenotype rather than that of non-parenchymal liver cells. For example, the HSC-associated genes such as those encoding α-smooth muscle actin (*ACTA2*, which is essential for the contractile phenotype of activated perisinusoidal cells) and fibroblast activation protein alpha (*FAP*, which is involved in tissue remodeling and fibrotic processes) were not detected at the transcript level. Other markers, such as desmin (*DES*, an intermediate filament characteristic of muscle and stellate cells), decorin (*DCN*, an extracellular matrix proteoglycan), glial fibrillary acidic protein (*GFAP*, an intermediate filament), and retinol binding protein 1 (*RBP1*, an intracellular retinoid transporter in stellate cells) were only found in trace amounts. The absence of these core stellate cell markers highlights the hepatocyte-like nature of HuH-7 cells (Supplementary Table S2).

Similarly, the following canonical Kupffer cell markers were either absent or present at extremely low levels in HuH-7 cells: *CD163* (a hemoglobin-haptoglobin scavenger receptor); C-type lectin domain family *member 1B (CLEC1B)* and member *CLEC4E* (both are pattern-recognition lectins); folate receptor β (*FOLR2*); Spi-C transcription factor (*SPIC*, which guides macrophage differentiation), Toll like receptor 9 (*TLR9*, which mediates innate immune responses via endosomes), *CLEC4G* (which is involved in pathogen recognition and immune regulation); myeloperoxidase (*MPO*, which is a hallmark enzyme in myeloid cells) (Supplementary Table S3). These proteins are key mediators of macrophage or immune cell activity, which further supports the idea that HuH-7 cells do not exhibit macrophage-like characteristics.

Finally, genes that are usually found in liver sinusoidal endothelial cells (LSECs), such as stabilin-1 (*STAB1*) and stabilin-2 (*STAB2*) (which are important for clearing lymph and blood waste), lymphatic vessel endothelial hyaluronan receptor-1 (*LYVE1*, a hyaluronan receptor found in lymphatic and sinusoidal endothelium), scavenger receptor class F member 1 (*SCARF1*, another scavenger receptor), and von Willebrand factor (*VWF*, a glycoprotein that plays a key role pivotal in platelet adhesion), were not expressed, or were only marginally expressed, in HuH-7 cells (Supplementary Table S4). These genes normally confer specialized endothelial functions that are absent in hepatocytes. Together, these results strongly support the conclusion that HuH-7 cells are of hepatocyte origin, further confirming their utility as a model for hepatic biology and liver carcinoma research.

### 3.11 Expression of hepatitis C virus host factors in HuH-7 cells

HuH-7 hepatoma cells and some of their derivatives, such as HuH-7.5, are the only continuous cell culture models that consistently support high-level replication of both sub-genomic replicons and fully infectious hepatitis C virus (Dächert et al., 2019). However, within this single lineage, the magnitude of viral RNA amplification varies significantly. Individual sub-clones or long-term passages can differ by up to three orders of magnitude in replication efficiency, highlighting the significant impact of subtle genetic or transcriptional drift on HCV permissiveness (Dächert et al., 2019). Specifically, seven host-dependency factors (*ZNF512B*, *SFI1*, *LBHD1*, *CRYM*, *CRAMP1*, *THAP7*, and NROB2) appear to play a crucial role in mediating HCV permissiveness (Dächert et al., 2019). In our bulk mRNA-sequencing analysis of our low-passage HuH-7 stock, we confirmed the expression of each of the HCV-permissiveness genes identified by Dächert and colleagues (Supplementary Table S5). This confirms that the molecular machinery previously associated with efficient HCV replication is intact in our HuH-7 cultures. Interestingly, we also detected the expression of THAP7 antisense RNA 1 (*THAP7-AS1*), a long noncoding RNA (lncRNA) that has been linked to oncogenic properties such as invasion and metastasis (Liu et al., 2022).

## 4 Discussion

HuH-7 cells, which were originally established in 1982 from human HCC tissue (Nakabayashi et al., 1982), have become one of the most prominent *in vitro* model systems for studying liver biology and disease. Since their introduction in the early 1980s, they have been widely used in biomedical research, particularly in areas such as viral hepatitis infection dynamics (Stuyver et al., 2003; Sainz et al., 2009; Padarath et al., 2024), drug metabolism (Choi et al., 2009; Bulutoglu et al., 2019; Li et al., 2022), aspects of cholestasis (Saran et al., 2022), and tumorigenesis (Chen et al., 2019; Mo and Wang, 2023). Thanks to their relatively straightforward cultivation and robust growth characteristics, these cells provide a cost-effective and reproducible platform for high-throughput assays. For example, research into hepatitis C virus replication has relied on HuH-7 cells to elucidate host-pathogen interactions and to screen antivirals prior to moving to clinical trials (Collett et al., 2021). Furthermore, HuH-7 cells have capacity to express numerous transporters relevant to drug metabolism (organic anion transporter polypeptides (OATPs), organic cation transporters (OCTs), multidrug resistance-associated proteins (MRPs), and many others, and produce and secrete various liver-specific proteins, making them indispensable for studies on lipid metabolism, iron homeostasis, and the toxicity testing of potential therapeutic compounds (Malinen et al., 2019; Mleczko-Sanecka et al., 2017; Jetter and Kullak-Ublick, 2020). Additionally, this cell line is used in xenograft models that facilitate preclinical tumor growth inhibition studies (Shao et al., 2019; Ye et al., 2025). Overall, the reliability and distinctive hepatic-like features of HuH-7 cells continue to underpin their broad adoption as a cornerstone model system in diverse areas of basic, translational, and preclinical research.

Our comprehensive analyses clearly show that HuH-7 cells have a phenotype consistent with a hepatocytic origin, rather than a non-parenchymal origin, in the liver. Firstly, the solid expression of classic hepatocyte markers (e.g., albumin, α-fetoprotein, and HNF4α) at both the transcript and protein levels clearly indicates a hepatocellular lineage. HuH-7 cells also express large quantities of keratin 19 (K19), a type I intermediate filament that is strongly implicated in the progression of various human carcinomas. By reinforcing cytoskeletal integrity and interacting with key signaling molecules, K19 promotes cancer cell survival, facilitates invasive behavior, and supports angiogenic processes that are essential for tumor expansion, particularly in the invasiveness of hepatocellular carcinomas (Takano et al., 2016; Govaere et al., 2014). This cytoskeletal protein therefore contributes to the aggressive phenotype of malignancies, making it a valuable marker and potential therapeutic target in hepatocellular carcinoma and other tumor types. Serpin A1, also known as α-1 antitrypsin, is robustly expressed in HuH-7 cells. This key acute-phase protein inhibits neutrophil elastase and other proteases. Under normal conditions, it maintains proteolytic balance and contributes significantly to immune regulation and inflammatory responses in the hepatic environment. Its capacity to regulate the proteolytic activity of enzymes that degrade extracellular matrices in the tumor environment may influence tumor progression and metastatic potential, which could explain the high activity of HuH-7 cells in xenograft models (Shao et al., 2019; Ye et al., 2025).

In our analysis, we observed that despite the relatively high quantities of FABP1 mRNA present, the corresponding FABP1 protein was either undetectable or exhibited very low levels in HuH-7 cells. This discrepancy may be attributed to several factors, including post-transcriptional regulation mechanisms such as microRNA-mediated repression, which can inhibit translation or promote mRNA degradation. Additionally, post-translational modifications or rapid protein degradation pathways could also contribute to the reduced levels of FABP1 protein detected in our Western blot analysis.

By contrast, markers that are specific to hepatic stellate cells (e.g., ACTA2, FAP, desmin, and retinol binding protein 1), Kupffer cells (e.g., CD163, CLEC1B, CLEC4E, FOLR2, SPIC, TLR9, and MPO), and liver sinusoidal endothelial cells (e.g., STAB1, STAB2, LYVE1, SCARF1, and von Willebrand factor) were either absent or present at negligible levels. Therefore, our findings reinforce that HuH-7 cells are derived from hepatocytes, corroborating the original description of this cell line as originating from a well-differentiated HCC.

Interestingly, we observed low expression of cytochrome P450 genes in HuH-7 cells, confirming a previous report (Bulutoglu et al., 2019). In primary hepatocytes, the expression of these genes is regulated by a network of transcription factors that respond to both endogenous signals and xenobiotic compounds (Ekiciler et al., 2023). At the core of this regulatory network are nuclear receptors such as the pregnane X receptor (PXR, encoded by the nuclear receptor subfamily 1 group I member 3, *NR1I2* gene) and the constitutive androstane receptor (*CAR*, encoded by the nuclear receptor subfamily 1 group I member 3 gene (*NR1I3*)). Upon ligand binding, these receptors form heterodimers with the retinoid X receptor (RXR) to activate *CYP* gene transcription. The aryl hydrocarbon receptor (*AHR*) is also involved, particularly in the induction of CYP1 family members after binding to environmental contaminants (Ye et al., 2019). Additionally, HNF4α plays a pivotal role in regulating the basal expression of several CYP enzymes (Jover et al., 2001). Together, these transcription factors ensure that cytochrome P450 levels adjust dynamically in response to metabolic states and the presence of potentially harmful substances. However, under the basal conditions analyzed, only AHR and HNF4α were found to be expressed at moderate levels (AHR: ΣTPM ∼48; HNF4α: ΣTPM ∼102), while the mRNA expression of NR1I3 (ΣTPM 0.05297) and NR1I2 (ΣTPM ∼3.3) was extremely low. Based on these findings, it is reasonable to conclude that NR1I3 and NR1I2 play a more significant role in regulating *CYP* genes in HuH-7 cells. Moreover, previous studies have shown that HuH-7 cells substantially induce *CYP3A4* mRNA, protein and activity when reaching confluency by endogenous induction of the pregnane X receptor (PXR) as a result of cell-cell contact, suggesting that factors dependent on culture-conditions are also relevant for the expression of *CYP* genes (Sivertsson et al., 2013). This suggests that these cells could be an ideal model for investigating the underlying mechanism of *CYP* gene regulation in future studies.

Another important finding is the presence of complex genomic rearrangements in HuH-7 cells, which could only be resolved through comprehensive molecular cytogenetic analyses. Overall, we identified similarities in certain translocations and copy numbers of chromosomes when compared with previously published SKY data (Kasai et al., 2018). However, our MCB study shows that neither M-FISH nor aCGH alone is able to provide insights into the karyotype of an advanced tumor cell line. The results of aCGH could be used to highlight that HuH-7 is indeed a suitable model for hepatocellular carcinoma. However, the relatively simple aCGH pattern provided no indication of the many complex rearrangements seen in MCB. This suggests that studying this and other cell lines using new approaches like optical genomic mapping (Paulraj et al., 2024) will lead to a better understanding of the influence of chromothriptic events in cell line evolution.

Kasai and colleagues showed that the HuH-7 hepatoma cell line exhibits extensive karyotypic diversity, with the number of chromosome typically clustering around 60, but ranging from 55 to 63 (Kasai et al., 2018). This is consistent with our finding that the cell line has an almost triploid karyotype. Multi-color fluorescence *in situ* hybridization (FISH) in the aforementioned study revealed abnormalities in every chromosome except chromosome 21, which appeared normal only by visual inspection. This chromosome was later found to exhibit copy-neutral loss of heterozygosity using SNP microarray. In our analysis, we confirmed that many chromosomes exhibit complex derivative forms. However, we also identified large deletions in chromosome 21. In line with the previous study, we confirmed the absence of the Y chromosome in HuH-7 cells. Complete loss of the Y chromosome (LOY) is frequently observed in cells originating from male tumors (Kido and Lau, 2015; Brown and Machiela, 2020). Recent studies have demonstrated that LOY, the most prevalent somatic alteration in men, is associated with aggressive cancer and poor prognosis (Abdel-Hafiz et al., 2023; Chen et al., 2025). Current research has revealed that LOY creates common dependencies on DEAD-box helicase 3 X-linked (*DDX3X*) and eukaryotic translation initiation factor 1A X-linked (*EIF1AX*) in male cell lines (Qi et al., 2023). These factors are abundantly expressed in HuH-7 cells. Therefore, HuH-7 cells may be useful for investigating how these two proteins can be targeted therapeutically, and the pathways through which they contribute to the multiple inter-chromosomal rearrangements, deletions, duplications, and pronounced genomic instability we observed in HuH-7 cells.

Our vCGH analysis of HuH-7 cells revealed several genomic alterations. Gains were identified on the short arms of chromosomes 3, 5, 18, and 20, and on the long arms of chromosomes 15, 16, and 20. Conversely, were detected losses on the short arms of chromosomes 5, 6, 8, 11, 12, and 17, and on the long arms of chromosomes 4, 8, 11, 12, 13, 14, 21, and 22. These genomic changes could potentially influence key cellular functions such as proliferation and apoptosis in HuH-7 cells. Therefore, it is crucial for researchers to consider these findings when conducting experiments involving HuH-7 cells, in order to accurately interpret the results and understand how the cells behave in relation to its genomic landscape.

The high level of heterogeneity observed in HuH-7 cells by us and others again underscores the importance of routine cell line characterization and quality control to ensure accurate and reproducible experimental results. In our view, this discrepancy in genetic findings underscores how ongoing subcloning, prolonged passage in culture, and other external factors can drive genomic evolution within the same cell line, resulting in divergence from earlier reference profiles. Despite these variations, our STR analysis showed a 100% match with the previously established STR profile for HuH-7 (Kasai et al., 2018). This finding that validates the identity of our current cell population, confirming that the observed alterations likely reflect clonal divergence over time rather than misidentification.

Our comprehensive (cyto)genetic and RNA-seq characterization complements and extends a previous functional study (Dächert et al., 2019). While their work focused on identifying transcriptomic differences that influence HCV permissiveness in various HuH-7 sub-clones, our data establishes a validated STR profile, resolves complex chromosomal rearrangements, and provides a high-resolution bulk-RNA expression atlas of a low-passage reference HuH-7 stock. Analysis of our RNA-seq dataset in conjunction with their candidate genes reveals that *THAP7* and NR0B2 are expressed at intermediate to high levels, while *CRYM*, *LBHD1,* and *CRAMP1* are present at lower but detectable amounts. This consistency suggests a cellular state that is inherently permissive to HCV but still responsive to further modulation. Importantly, we confirm robust expression of the classical HCV-entry receptors CD81, SCARB1, CLDN1 and OCLN at the mRNA level, confirming the suitability of our HuH-7 cells for virus-host interaction studies. The previous study by Dächert and colleagues highlights specific transcriptional determinants of viral replication, while our work provides an authenticated genomic framework and transcript abundance reference that will aid in dissecting how these, and newly emerging, host factors interact with the complex karyotype of HuH-7 cells to influence HCV biology.

In light of the significant heterogeneity reported in numerous studies, including those documented here, it is crucial for laboratories to consistently authenticate and characterize their cell lines at the genetic level (Horbach and Halffman, 2017; Souren et al., 2022; Weiskirchen et al., 2023). Routine short tandem repeat (STR) profiling and molecular analyses help to detect possible cross-contamination, confirm cell line identity, and track genetic drift, all of which could influence experimental outcomes (Horbach and Halffman, 2017; Souren et al., 2022; Weiskirchen et al., 2023).

In addition to commercial STR kits, several low-cost assays can be incorporated into the routine workflow to confirm HuH-7 identity before extensive experimentation. (i) A rapid multiplex PCR for species- and sex-determining loci (e.g., amelogenin) can rule out cross-species contamination within 2 hours. (ii) HuH-7 cells carry a stable TP53 Y220C mutation (Tseng et al., 2022) and lack the Y chromosome. Both charactersitics can be confirmed through Sanger sequencing or a single PCR that detects the p53 codon-220 variant along with an SRY amplicon. (iii) Our bulk RNA-seq data demonstrates very high expression of albumin, α-fetoprotein (*AFP*), hepatocyte-nuclear-factor-4α (*HNF4A*) and keratin-19 (*KRT19*), along with minimal levels of hepatic stellate cell (*ACTA2*) or Kupffer cell (*CD163*) markers. A simple four-gene qPCR or Western blot panel (ALB, AFP, HNF4A, and KRT19) can reliably differentiate authentic HuH-7 from other hepatic or non-hepatic cell lines. These PCR/Western assays can be conducted using standard reagents and equipment, offering a practical alternative or rapid supplement to full STR profiling for daily cell line authentication.

**FIGURE 10:**
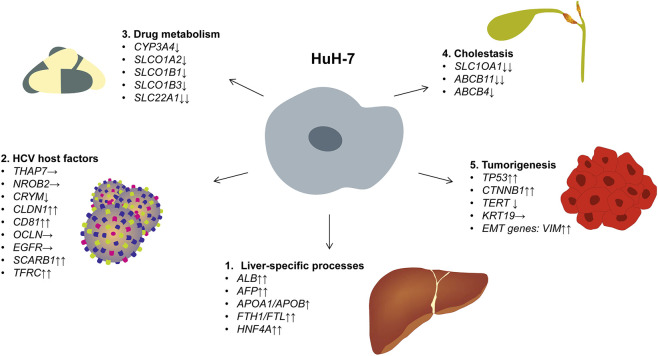
Gene expression in HuH-7 cells. The figure illustrates the expression of representative genes associated with liver-specific functions, hepatitis C virus permissiveness, drug metabolism, cholestasis, and tumorigenesis. Based on our bulk mRNA sequencing data, expression levels are categorized as follows: ↑↑ very high expression (TPM >100), ↑ high expression (TPM 50–100), → moderate expression (TPM 10–50), ↓ low expression (TPM 1–10), and ↓↓ very low/no expression (TPM <1). The complete mRNA expression data of HuH-7 cells, including exact TPM values for each gene, are depicted in Supplementary Table S1.

In the context of HuH-7, our transcriptomic data also provide a valuable resource for researchers seeking to use to study tumor formation, drug metabolism, or viral infection. Notably, we identified transcripts and proteins that are crucial for liver-specific processes, such as iron storage (ferritin chain proteins), lipoprotein metabolism (apolipoproteins), and the acute-phase response proteins (e.g., α1-antitrypsin). These expression patterns further validate the utility of HuH-7 for investigating hepatic functions and pathophysiological pathways relevant to HCC. HuH-7 cells express a large number of genes that are specific for liver, HCV permissiveness, drug metabolism, cholestasis, and tumorigenesis (Figure 10). The reference atlas presented here will serve as a guide for the rational design of subsequent *in vivo* studies. This will enable researchers to choose genomically characterized HuH-7 sub-clones and develop xenograft experiments that investigate the functional significance of particular alterations within a physiological setting. Additionally, our expression data will provide the research community with the opportunity to conduct systematic analysis using GO, KEGG, and GSEA methods, potentially revealing further pathway enrichments and functional networks that regulate HuH-7 biology. This will further enhance the value of the molecular profile outlined in this study.

Despite the high overall concordance that we observed with historical karyotypes and STR data, HuH-7 must be regarded as a dynamic, evolving population in which passage number, culture conditions and sporadic sub-cloning can all create measurable genetic and phenotypic drift. To minimize inter-laboratory variability we recommend a two-tier, “same-day” authentication workflow that laboratories can routinely apply before embarking on extensive experimentation. Firstly, a rapid multiplex PCR that amplifies the amelogenin sex locus together with the TP53 c.659A>G (p.Y220C) hotspot, present in all *bona fide* HuH-7 stocks, confirms both species origin and the signature tumor mutation within 2 hours. Secondly, a downstream four-marker expression panel, implemented either as qPCR or Western blot, interrogates albumin (ALB), α-fetoprotein (AFP), hepatocyte-nuclear-factor-4α (HNF4A) and keratin-19 (KRT19). These genes are more than 50-fold enriched in our bulk-RNA dataset, whereas transcripts typical of hepatic stellate cells (*ACTA2*), Kupffer cells (*CD163*) or LSEC (*STAB1/2*) are virtually absent (Supplementary Tables S1–S4). Nevertheless, it might be possible that the expression pattern is slightly different in other HuH-7 sub-clones or in batches with different passage numbers, which might reflect clonal drift and passage-related adaptations. Therefore, this underscores the need for each laboratory to verify key markers in its own stocks before undertaking critical experiments.

Together, the mutation/sex PCR and the four-marker hepatocyte panel can be completed with standard reagents in a single working day and provide an inexpensive yet robust benchmark against the molecular portrait presented here, allowing individual laboratories to detect cross-contamination, monitor clonal drift and harmonize their HuH-7 sub-clones with the reference stock characterized in this study.

## 5 Conclusion

Our analyses confirmed the authenticity and hepatocytic origin of HuH-7, while revealing genetic alterations indicative of its tumor-derived background. Short tandem repeat profiling verified the identity of the cell line, and spectral karyotyping revealed complex chromosomal rearrangements and aneuploidy. Expression studies on mRNA and protein levels confirmed the expression of key liver-specific markers such as albumin, α-fetoprotein, and HNF4α, and excluded the presence of non-parenchymal liver cells. These findings emphasize the value of the HuH-7 cell line in studying liver carcinogenesis, hepatic functions, drug metabolism, and viral infections. However, the observed genetic and phenotypic variability underscores the importance of routine authentication and molecular characterization to ensure reliable and reproducible research.

## Data Availability

The original contributions presented in the study are included in the article/Supplementary Material, further inquiries can be directed to the corresponding authors.
