# Supplementary material for: Decoding HuH-7: a comprehensive genetic and molecular portrait of a widely used hepatocellular carcinoma model
Source: Front Cell Dev Biol. 2025 Nov 11;13:1648639. doi: 10.3389/fcell.2025.1648639 (PMC12643976; doi:10.3389/fcell.2025.1648639)
Supplement: Supplementary file 1 [file DataSheet1.pdf]

Supplementary Material

Supplementary Tables

Supplementary Table 2: Expression of selected hepatic stellate cell-specific genes in HuH-7 cells

Gene	Gene description	Gene Id	Transcript Id[1]	TPM[1]
ACTA2	actin alpha 2, smooth muscle	ENSG00000107796	ND	0
BGN	biglycan	ENSG00000182492	ENST00000331595.9	0.494072
CCL2	C-C motif chemokine ligand 2	ENSG00000108691	ENST00000225831.4	0.117459
COL1A2	collagen type I alpha 2 chain	ENSG00000164692	ENST0000297268.11	0.19271
COL3A1	collagen type III alpha 1 chain	ENSG00000168542	ENST00000304636.9	0.773738
COLEC11	collectin subfamily member 11	ENSG00000118004	ND	0
DCN	decorin	ENSG00000011465	ENST00000441303.6	0.191277
DES	desmin	ENSG00000175084	ENST00000373960.4	0.13006
FAP	fibroblast activation protein alpha	ENSG00000078098	ND	0
FGF10	fibroblast growth factor 10	ENSG00000070193	ND	0
GFAP	glial fibrillary acidic protein	ENSG00000131095	ENST00000253408.11	0.045202
MEG3	maternally expressed 3	ENSG00000214548	ND	0
NGFR	nerve growth factor receptor	ENSG00000064300	ENST00000172229.8	0.249964
RBP1	retinol binding protein 1	ENSG00000114115	ENST00000483943.6<br>ENST00000492918.1	0.316063<br>0.191381
SEMA7A	semaphorin 7A	ENSG00000138623	ENST00000261918.9<br>ENST00000542748.6	0.443048<br>0.254762
SLC8A1	solute carrier family 8 member A1	ENSG00000183023	ENST00000406391.2	0.025503
TIMP1	TIMP metallopeptidase inhibitor 1	ENSG00000102265	ENST00000218388.9	1.344057
WT1	WT1 transcription factor	ENSG00000184937	ND	0
ACTB	actin beta	ENSG00000075624	ENST00000646664.1<br>ENST00000676397.1<br>ENST00000493945.6<br>ENST00000432588.6<br>ENST00000642480.2<br>ENST00000473257.3<br>ENST00000675515.1	3716.97217<br>33.690344<br>23.548124<br>6.407391<br>5.493343<br>2.582519<br>0.164191
GAPDH	glyceraldehyde-3-phosphate dehydrogenase	ENSG00000111640	ENST00000229239.10<br>ENST00000396856.5<br>ENST00000396859.5<br>ENST00000619601.1<br>ENST00000396861.5<br>ENST00000396858.5	5285.775364<br>31.631692<br>31.531195<br>16.213084<br>15.90503<br>0.202033

[1] For a comparison of the transcript levels of the listed genes, the expressions of actin beta (*ACTB*) and glyceraldehyde-3-phosphate dehydrogenase (*GAPDH*) are depicted. The complete mRNA expression profile of HuH-7 cells, as observed by NGS, can be found in Table S1. Abbreviations used: ND, no transcripts of this gene were detected; TPM, Transcripts Per Million.

Supplementary Table 3: Expression of selected Kupffer cell-specific genes in HuH-7 cells

Gene	Gene description	Gene Id	Transcript Id[1]	TPM[1]
ADGRE1	adhesion G protein-coupled receptor E1	ENSG00000174837	ND	0
C1QA	complement C1q A chain	ENSG00000173372	ND	0
C1QB	complement C1q B chain	ENSG00000173369	ND	0
C1QC	complement C1q C chain	ENSG00000159189	ND	0
CCR5	C-C motif chemokine receptor 5	ENSG00000160791	ENST00000292303.5	0.021157
CD14	CD14 molecule	NSG00000170458	ENST00000302014.11<br>ENST00000512545.2	1.113186<br>0.154393
CD163	CD163 molecule	ENSG00000177575	ND	0
CHIT1	chitinase 1	ENSG00000133063	ND	0
CLEC1B	C-type lectin domain family 1 member B	ENSG00000165682	ND	0
CLEC4E	C-type lectin domain family 4 member E	ENSG00000166523	ND	0
CLEC4F	C-type lectin domain family 4 member F	ENSG00000152672	ENST00000426626.1	0.548761
CLEC4G	C-type lectin domain family 4 member G	ENSG00000182566	ND	0
CSF1R	colony stimulating factor 1 receptor	ENSG00000182578	ND	0
DNASE1L3	deoxyribonuclease 1 like 3	ENSG00000163687	ND	0
FCN1	ficolin 1	ENSG00000085265	ND	0
FOLR2	folate receptor beta	ENSG00000165457	ND	0
GPIHBP1	glycosylphosphatidylinositol anchored high density lipoprotein binding protein 1	ENSG00000277494	ND	0
IL1B	interleukin 1 beta	ENSG00000125538	ENST00000263341.7	0.050363
IRF7	interferon regulatory factor 7	ENSG00000185507	ENST00000525445.6	0.115312
MARCO	macrophage receptor with collagenous structure	ENSG00000019169	ENST00000327097.5	0.164318
MNDA	myeloid cell nuclear differentiation antigen	ENSG00000163563	ND	0
MPO	myeloperoxidase	ENSG00000005381	ND	0
MSR1	macrophage scavenger receptor 1	ENSG00000038945	ENST00000355282.6	0.067471
OSM	oncostatin M	ENSG00000099985	ND	0
PROK2	prokineticin 2	ENSG00000163421	ENST00000353065.7	0.048833
SLC11A1	solute carrier family 11 member 1	ENSG00000018280	ENST00000233202.11	0.196199
SLC15A3	solute carrier family 15 member 3	ENSG00000110446	ENST00000227880.8	0.115441
SPIC	Spi-C transcription factor	ENSG00000166211	ND	0
STARD5	StAR related lipid transfer domain containing 5	ENSG00000172345	ENST00000302824.7	0.157348
TIMD4	T cell immunoglobulin and mucin domain containing 4	ENSG00000145850	ENST00000274532.7<br>ENST00000407087.4	1.765135<br>0.246839
TLR4	toll like receptor 4	ENSG00000136869	ENST00000355622.8	0.005398
TLR9	toll like receptor 9	ENSG00000239732	ND	0
TNF	tumor necrosis factor	ENSG00000232810	ND	0

TREM1	triggering receptor expressed on myeloid cells 1	ENSG00000124731	ENST00000591620.1	0.071768
VSIG4	V-set and immunoglobulin domain containing 4	ENSG00000155659	ND	0
ACTB	actin beta	ENSG00000075624	ENST00000646664.1<br>ENST00000676397.1<br>ENST00000493945.6<br>ENST00000432588.6<br>ENST00000642480.2<br>ENST00000473257.3<br>ENST00000675515.1	3716.97217<br>33.690344<br>23.548124<br>6.407391<br>5.493343<br>2.582519<br>0.164191
GAPDH	glyceraldehyde-3-phosphate dehydrogenase	ENSG00000111640	ENST00000229239.10<br>ENST00000396856.5<br>ENST00000396859.5<br>ENST00000619601.1<br>ENST00000396861.5<br>ENST00000396858.5	5285.775364<br>31.631692<br>31.531195<br>16.213084<br>15.90503<br>0.202033

[1] For a comparison of the transcript levels of the listed genes, the expressions of actin beta *(ACTB*) and glyceraldehyde-3-phosphate dehydrogenase (*GAPDH*) are depicted. The complete mRNA expression profile of HuH-7 cells, as observed by NGS, can be found in Table S1. Abbreviations used: ND, no transcripts of this gene were detected; TPM, Transcripts Per Million.

Supplementary Table 4: Expression of selected liver sinusoidal endothelial cell-specific genes in HuH-7 cells

Gene	Gene description	Gene Id	Transcript Id[1]	TPM[1]
AOC3	amine oxidase copper containing 3	ENSG00000131471	ENST00000308423.7<br>ENST00000591562.1<br>ENST00000613571.1	2.152991<br>1.621233<br>0.330603
FCGR2B	Fc gamma receptor IIb	ENSG00000072694	ENST00000236937.13	0.106023
LYVE1	lymphatic vessel endothelial hyaluronan receptor 1	ENSG00000133800	ENST00000256178.8	0.043838
MADCAM1	mucosal vascular addressin cell adhesion molecule 1	ENSG00000099866	ENST00000587541.5<br>ENST00000382683.8<br>ENST00000215637.8	0.416069<br>0.18185<br>0.158698
PECAM1	platelet and endothelial cell adhesion molecule 1	ENSG00000261371	ENST00000563924.6	4.8969
STAB1	stabilin 1	ENSG00000010327	ENST00000321725.10	0.0087
STAB2	stabilin 2	ENSG00000136011	ENST00000388887.7	0.016722
SCARF1	scavenger receptor class F member 1	ENSG00000074660	ND	0
VWF	von Willebrand factor	ENSG00000110799	ENST00000261405.10	0.007794
ACTB	actin beta	ENSG00000075624	ENST00000646664.1<br>ENST00000676397.1<br>ENST00000493945.6<br>ENST00000432588.6<br>ENST00000642480.2<br>ENST00000473257.3<br>ENST00000675515.1	3716.97217<br>33.690344<br>23.548124<br>6.407391<br>5.493343<br>2.582519<br>0.164191
GAPDH	glyceraldehyde-3-phosphate dehydrogenase	ENSG00000111640	ENST00000229239.10<br>ENST00000396856.5<br>ENST00000396859.5<br>ENST00000619601.1<br>ENST00000396861.5<br>ENST00000396858.5	5285.775364<br>31.631692<br>31.531195<br>16.213084<br>15.90503<br>0.202033

[1] For a comparison of the transcript levels of the listed genes, the expressions of actin beta *(ACTB*) and glyceraldehyde-3-phosphate dehydrogenase (*GAPDH*) are depicted. The complete mRNA expression profile of HuH-7 cells observed by NGS can be found in Table S1. Abbreviations used: ND, no transcripts of this gene were detected; TPM, Transcripts Per Million.

Supplementary Table 5: Expression of predicted hepatitis C virus host factors in HuH-7 cells

Gene[1]	**Gene description**	**Gene Id**	**Transcript Id**[2]	**TPM**[1]
CRAMP1	cramped chromatin regulator homolog 1	ENSG00000007545	ENST00000293925.9<br>ENST00000397412.8	5.049515<br>1.955733
CRYM	crystallin mu	ENSG00000103316	ENST00000572914.2	5.262695
LBHD1 (C11orf48)	LBH domain containing 1	ENSG00000162194	ENST00000431002.6<br>ENST00000354588.8	1.796372<br>1.650259
NR0B2 (*SHP)*	nuclear receptor subfamily 0 group B member 2	ENSG00000131910	ENST00000254227.4	24.135425
SFI1	SFI1 centrin binding protein	ENSG00000198089	ENST00000400288.7<br>ENST00000400289.5<br>ENST00000540643.5<br>ENST00000443011.5	3.755793<br>1.18876<br>1.029087<br>0.698115
THAP7	THAP domain containing 7	ENSG00000184436	ENST00000399133.2<br>ENST00000215742.9	25.595885<br>2.603859
THAP7-AS1	THAP7 antisense RNA 1	ENSG00000230513	ENST00000436079.1<br>ENST00000452284.1	2.301925<br>0.159401
ZNF512B	zinc finger protein 512B	ENSG00000196700	ENST00000369888.6	1.962291
ACTB	actin beta	ENSG00000075624	ENST00000646664.1<br>ENST00000676397.1<br>ENST00000493945.6<br>ENST00000432588.6<br>ENST00000642480.2<br>ENST00000473257.3<br>ENST00000675515.1	3716.97217<br>33.690344<br>23.548124<br>6.407391<br>5.493343<br>2.582519<br>0.164191
GAPDH	glyceraldehyde-3-phosphate dehydrogenase	ENSG00000111640	ENST00000229239.10<br>ENST00000396856.5<br>ENST00000396859.5<br>ENST00000619601.1<br>ENST00000396861.5<br>ENST00000396858.5	5285.775364<br>31.631692<br>31.531195<br>16.213084<br>15.90503<br>0.202033

[1] In previous knock-down studies, the genes CRAMP1, CRYM, LBHD1, NR0B2, SFI1, THAP7, and ZNF512B showed implications in HCV replication (Dächert et al., 2020). [2] For a comparison of the transcript levels of the listed genes, the expressions of actin beta *(ACTB*) and glyceraldehyde-3-phosphate dehydrogenase (*GAPDH*) are depicted. The complete mRNA expression profile of HuH-7 cells observed by NGS can be found in Table S1. Abbreviation used: TPM, Transcripts Per Million.

Supplementary Table 6: Selected gene expression in HuH-7 cells that underpins their hepatocytic origin[1]

Gene	Gene description	Gene Id	Transcript Id	TPM[2]
ABCC3	ATP binding cassette subfamily C member 3	ENSG00000108846	ENST00000285238.13<br>ENST00000427699.5<br>ENST00000515707.1	5.411044<br>1.108026<br>0.275668
ABCD3	ATP binding cassette subfamily D member 3	ENSG00000117528	ENST00000370214.9<br>ENST00000315713.5	109.991235<br>17.535516
ACADM	acyl-CoA dehydrogenase medium chain	ENSG00000117054	ENST00000370841.9<br>ENST00000420607.6<br>ENST00000680964.1<br>ENST00000680805.1<br>ENST00000679687.1	57.964581<br>38.1719<br>1.343853<br>0.984129<br>0.639576
ACAT2	acetyl-CoA acetyltransferase 2 [	ENSG00000120437	ENST00000367048.5	490.01092
ACLY	ATP citrate lyase	ENSG00000131473	ENST00000352035.7<br>ENST00000353196.5<br>ENST00000590151.5<br>ENST00000393896.6	145.333931<br>86.238545<br>74.594288<br>57.214373
ACSS2	acyl-CoA synthetase short chain family member 2	ENSG00000131069	ENST00000360596.7<br>ENST00000253382.5	52.484305<br>0.241686
AFP	alpha fetoprotein	ENSG00000081051	ENST00000395792.7<br>ENST00000226359.2	1068.981533<br>784.704493
AHR	aryl hydrocarbon receptor	ENSG00000106546	ENST00000242057.9<br>ENST00000642825.1	46.985221<br>1.09226
A1CF	APOBEC1 complementation factor	ENSG00000148584	ENST00000374001.6<br>ENST00000373993.6<br>ENST00000373997.8<br>ENST00000395489.7<br>ENST00000373995.7	26.589698<br>14.764128<br>3.52316<br>1.437412<br>0.155239
AKR1C1	aldo-keto reductase family 1 member C1	ENSG00000187134	ENST00000380859.1<br>ENST00000380872.9	4.137038<br>2.082184
ALB	albumin	ENSG00000163631	ENST00000509063.5<br>ENST00000415165.6<br>ENST00000295897.9<br>ENST00000401494.7<br>ENST00000503124.5	5026.854128<br>2410.505634<br>1856.352717<br>11.497756<br>3.287102
ALDH6A1	aldehyde dehydrogenase 6 family member A1	ENSG00000119711	ENST00000553458.6<br>ENST00000350259.8<br>ENST00000555126.1	9.989024<br>8.117909<br>0.495415
AMBP	alpha-1-microglobulin/bikunin precursor	ENSG00000106927	ENST00000265132.8	1004.193092
ANG	angiogenin	ENSG00000214274	ENST00000336811.10<br>ENST00000397990.5	19.245275<br>5.390933
ANXA13	annexin A13	ENSG00000104537	ENST00000419625.6<br>ENST00000262219.10	2.017452<br>1.684361
APOA1	apolipoprotein A1	ENSG00000118137	ENST00000236850.5<br>ENST00000375323.5<br>ENST00000359492.6<br>ENST00000375320.5<br>ENST00000375329.6	479.580739<br>41.49574<br>19.352689<br>6.12056<br>5.409424

APOA2	apolipoprotein A2	ENSG00000158874	ENST00000367990.7<br>ENST00000468465.5<br>ENST00000463812.1<br>ENST00000464492.5	851.641601<br>23.30225<br>19.396845<br>2.461307
APOA5	apolipoprotein A5	ENSG00000110243	ENST00000227665.9	0.551303
APOB	apolipoprotein B	ENSG00000084674	ENST00000233242.5<br>ENST00000399256.4	418.665918<br>0.770799
APOC3	apolipoprotein C3	ENSG00000110245	ENST00000227667.8	101.38132
APOH	apolipoprotein H	ENSG00000091583	ENST00000205948.11	97.622672
APOM	apolipoprotein M	ENSG00000204444	ENST00000375916.4<br>ENST00000375918.6	40.482114<br>1.551169
AQP3	aquaporin 3 (Gill blood group)	ENSG00000165272	ENST00000297991.6	28.9842
ARG1	arginase 1	ENSG00000118520	ENST00000368087.8<br>ENST00000356962.2<br>ENST00000673427.1	27.344412<br>0.562976<br>0.229806
ASGR1	asialoglycoprotein receptor 1	ENSG00000141505	ENST00000269299.8<br>ENST00000619926.4<br>ENST00000572879.5<br>ENST00000574388.5	25.499817<br>4.861638<br>1.92106<br>1.385759
ASL	argininosuccinate lyase	ENSG00000126522	ENST00000395332.8<br>ENST00000673518.1<br>ENST00000380839.9<br>ENST00000304874.14<br>ENST00000395331.4	39.212551<br>21.491007<br>6.070932<br>4.366237<br>0.314354
ASS1	argininosuccinate synthase 1	ENSG00000130707	ENST00000372394.5<br>ENST00000372393.7<br>ENST00000352480.10	400.899918<br>22.238217<br>0.793815
ATP7B	ATPase copper transporting beta	ENSG00000123191	ENST00000242839.10<br>ENST00000673772.1<br>ENST00000448424.7	6.866589<br>0.866004<br>0.524998
BNIP3	BCL2 interacting protein 3	ENSG00000176171	ENST00000368636.9<br>ENST00000633835.2<br>ENST00000540159.4	308.766968<br>10.429135<br>6.933424
C1ORF53	chromosome 1 open reading frame 53	ENSG00000203724	ENST00000367393.8	5.135328
C4B	complement C4B	ENSG00000224389	ENST00000435363.7	4.643549
CDH1	cadherin 1	ENSG00000039068	ENST00000261769.10<br>ENST00000422392.6	53.193162<br>0.905931
CEBPA	CCAAT enhancer binding protein alpha	ENSG00000245848	ENST00000498907.3	283.638502
CP	ceruloplasmin	ENSG00000047457	ENST00000264613.11	11.108003
CPS1	carbamoyl-phosphate synthase 1	ENSG00000021826	ENST00000233072.10<br>ENST00000673510.1	0.608039<br>0.467479
CRP	C-reactive protein	ENSG00000132693	ND	0
CTNNB1	catenin beta 1	ENSG00000168036	ENST00000396185.8<br>ENST00000646725.1<br>ENST00000645982.1<br>ENST00000643031.1<br>ENST00000647390.1<br>ENST00000643992.1<br>ENST00000642886.1<br>ENST00000643297.1<br>ENST00000647264.1<br>ENST00000642426.1<br>ENST00000645276.1<br>ENST00000644873.1	74.324211<br>63.255902<br>15.640361<br>13.958452<br>11.670908<br>4.461679<br>3.900712<br>3.194732<br>2.363529<br>2.238538<br>2.12679<br>1.31504

			ENST00000647413.2<br>ENST00000644138.1<br>ENST00000645320.1<br>ENST00000431914.6<br>ENST00000644867.1<br>ENST00000646174.1<br>ENST00000644524.1<br>ENST00000645493.1	0.899867<br>0.695768<br>0.531144<br>0.420068<br>0.271178<br>0.227283<br>0.154312<br>0.139239
CYP1A1	cytochrome P450 family 1 subfamily A member 1	ENSG00000140465	ENST00000379727.8<br>ENST00000395048.6	7.357747<br>1.700431
CYP1A2	cytochrome P450 family 1 subfamily A member 2	ENSG00000140505	ENST00000343932.5	0.022769
CYP2A6	cytochrome P450 family 2 subfamily A member 6	ENSG00000255974	ENST00000301141.10	0.083751
CYP2A7	cytochrome P450 family 2 subfamily A member 7	ENSG00000198077	ENST00000301146.9	0.08567
CYP2B6	cytochrome P450 family 2 subfamily B member 6	ENSG00000197408	ENST00000324071.10<br>ENST00000593831.1	4.007195<br>2.121862
CYP2C8	cytochrome P450 family 2 subfamily C member 8	ENSG00000138115	ENST00000535898.5	0.248138
CYP2C9	cytochrome P450 family 2 subfamily C member 9	ENSG00000138109	ENST00000260682.8	0.366568
CYP2C19	cytochrome P450 family 2 subfamily C member 19	ENSG00000165841	ENST00000371321.9	0.136255
CYP2D6	cytochrome P450 family 2 subfamily D member 6	ENSG00000100197	ENST00000359033.4	0.676141
CYP2E1	cytochrome P450 family 2 subfamily E member 1	ENSG00000130649	ND	0
CYP3A4	cytochrome P450 family 3 subfamily A member 4	ENSG00000160868	ND	0
CYP3A7	cytochrome P450 family 3 subfamily A member 7	ENSG00000160870	ND	0
CYP7A1	cytochrome P450 family 7 subfamily A member 1	ENSG00000167910	ND	0
DEFB1	defensin beta 1	ENSG00000164825	ENST00000297439.4	15.657857
ENTPD5	ectonucleoside triphosphate diphosphohydrolase 5 (inactive)	ENSG00000187097	ENST00000334696.11<br>ENST00000557325.5<br>ENST00000556242.5	52.760183<br>3.30735<br>0.951792
EPB41L4B	erythrocyte membrane protein band 4.1 like 4B	ENSG00000095203	ENST00000374557.4<br>ENST00000374566.8	12.995235<br>0.130356
EPPK1	epiplakin 1	ENSG00000261150	ENST00000615648.2	5.282482
FABP1	fatty acid binding protein 1	ENSG00000163586	ENST00000295834.8<br>ENST00000393750.3	1022.282132<br>1.866548
FGA	fibrinogen alpha chain	ENSG00000171560	ENST00000403106.8<br>ENST00000651975.2	804.238787<br>67.580784
FGB	fibrinogen beta chain	ENSG00000171564	ENST00000509493.1<br>ENST00000302068.9	723.333107<br>224.64291
FGG	fibrinogen gamma chain	ENSG00000164687	ENST00000404648.7<br>ENST00000407946.5<br>ENST00000405164.5<br>ENST00000336098.8	373.801269<br>59.853934<br>40.068397<br>8.820809
FGL1	fibrinogen like 1	ENSG00000104760	ENST00000427924.5<br>ENST00000518650.5<br>ENST00000381841.4<br>ENST00000398056.6	27.820338<br>8.833352<br>4.253756<br>0.806466

FGFR4	fibroblast growth factor receptor 4	ENSG00000160867	ENST00000292408.9<br>ENST00000502906.5<br>ENST00000393648.6<br>ENST00000393637.5	157.688635<br>69.280419<br>20.030395<br>12.297557
FN1	fibronectin 1	ENSG00000115414	ENST00000354785.11<br>ENST00000443816.5<br>ENST00000446046.5<br>ENST00000323926.10<br>ENST00000432072.6<br>ENST00000426059.1	445.829638<br>222.634594<br>132.240541<br>48.233483<br>31.046218<br>1.288296
FOXA1	forkhead box A1	ENSG00000129514	ENST00000250448.5	33.614447
FOXA2	forkhead box A2	ENSG00000125798	ENST00000377115.4<br>ENST00000419308.7	61.98258<br>26.298633
FOXA3	forkhead box A3	ENSG00000170608	ENST00000302177.3	29.942726
FST	follistatin	ENSG00000134363	ENST00000256759.8<br>ENST00000396947.7	16.315592<br>3.179729
FTH1	ferritin heavy chain 1	ENSG00000167996	ENST00000620041.5<br>ENST00000273550.12<br>ENST00000526640.5<br>ENST00000529631.5<br>ENST00000532601.1<br>ENST00000529191.5	3617.934969<br>2430.160321<br>468.800171<br>7.988079<br>5.402953<br>0.753325
FTL	ferritin light chain	ENSG00000087086	ENST00000331825.11	4436.265555
G0S2	G0/G1 switch 2	ENSG00000123689	ENST00000367029.5	0.85612
GC	GC vitamin D binding protein	ENSG00000145321	ENST00000273951.13	1.911797
GCK	glucokinase	ENSG00000106633	ND	0
GHR	growth hormone receptor	ENSG00000112964	ENST00000230882.9<br>ENST00000537449.5	1.556932<br>1.50656
GJB2	gap junction protein beta 2	ENSG00000165474	ENST00000382848.5	1.780546
GLS2	glutaminase 2	ENSG00000135423	ENST00000623608.3<br>ENST00000311966.9	0.336513<br>0.221366
GPAT4	glycerol-3-phosphate acyltransferase 4	ENSG00000158669	ENST00000396987.7	33.732265
GPC3	glypican 3	ENSG00000147257	ENST00000370818.8<br>ENST00000631057.2<br>ENST00000689310.1<br>ENST00000394299.7	567.58236<br>29.821612<br>19.307112<br>6.864262
GLUL	glutamate-ammonia ligase	ENSG00000135821	ENST00000339526.8<br>ENST00000331872.11<br>ENST00000417584.6<br>ENST00000311223.9	174.690605<br>14.633245<br>6.081557<br>0.359024
GOLT1A	golgi transport 1A	ENSG00000174567	ENST00000308302.4	17.050775
GRB14	growth factor receptor bound protein 14	ENSG00000115290	ENST00000263915.8<br>ENST00000696453.2	7.605801<br>5.64976
GRP	gastrin releasing peptide	ENSG00000134443	ND	0
GSTA2	glutathione S-transferase alpha 2	ENSG00000244067	ENST00000493422.3	0.320086
HAL	histidine ammonia-lyase	ENSG00000084110	ENST00000261208.8<br>ENST00000538703.5<br>ENST00000541929.5	12.438387<br>10.538754<br>1.360067
HAMP	hepcidin antimicrobial peptide	ENSG00000105697	ENST00000222304.5	0.559711
HMGCS1	3-hydroxy-3-methylglutaryl-CoA synthase 1	ENSG00000112972	ENST00000433297.2<br>ENST00000325110.11	754.36224<br>35.582875
HNF4A	hepatocyte nuclear factor 4 alpha	ENSG00000101076	ENST00000316673.9<br>ENST00000316099.10	48.552653<br>40.712645

			ENST00000415691.2<br>ENST00000443598.6	10.090812<br>2.145697
HP	haptoglobin	ENSG00000257017	ENST00000355906.10<br>ENST00000398131.6	6.426395<br>2.581071
HPR	haptoglobin-related protein	ENSG00000261701	ENST00000540303.7	3.849199
KIF13B	kinesin family member 13B	ENSG00000197892	ENST00000524189.6<br>ENST00000521515.1	14.885443<br>1.387584
KRT8	keratin 8	ENSG00000170421	ENST00000692008.1<br>ENST00000552150.5<br>ENST00000552551.5<br>ENST00000619952.2<br>ENST00000293308.11	3054.08644199999<br>26.824888<br>25.416126<br>2.679175<br>2.174108
KRT18	keratin 18	ENSG00000111057	ENST00000388835.4<br>ENST00000388837.6<br>ENST00000550600.5	2392.850981<br>38.581545<br>4.101943
KRT19	keratin 19	ENSG00000171345	ENST00000361566.7	23.284769
LEPR	leptin receptor	ENSG00000116678	ENST00000371060.7<br>ENST00000616738.4<br>ENST00000371059.7<br>ENST00000371058.1<br>ENST00000349533.11	5.233358<br>3.527942<br>2.285827<br>0.812115<br>0.22691
LIPC	lipase C, hepatic type	ENSG00000166035	ENST00000588188.7<br>ENST00000356113.10<br>ENST00000299022.10<br>ENST00000414170.7	79.911997<br>28.699433<br>11.889459<br>0.852596
LRP5	LDL receptor related protein 5<br>[	ENSG00000162337	ENST00000294304.12	140.209953
MCC	MCC regulator of WNT signaling pathway	ENSG00000171444	ENST00000302475.9<br>ENST00000514701.5	11.382496<br>0.182403
MSMO1	methylsterol monooxygenase 1	ENSG00000052802	ENST00000261507.11<br>ENST00000393766.6<br>ENST00000504317.1	287.11002<br>29.949633<br>2.293034
NOS2	nitric oxide synthase 2	ENSG00000007171	ENST00000697339.1	0.074539
OCIAD1	OCIA domain containing 1	ENSG00000109180	ENST00000513391.2<br>ENST00000396448.6<br>ENST00000381473.7<br>ENST00000264312.12<br>ENST00000508293.5<br>ENST00000444354.6	33.816391<br>8.933271<br>3.947959<br>1.77301<br>0.29131<br>0.190061
ORM1	orosomucoid 1	ENSG00000229314	ENST00000259396.9	173.756453
OTC	ornithine transcarbamylase	ENSG00000036473	ENST00000039007.5	0.176958
PAH	phenylalanine hydroxylase	ENSG00000171759	ENST00000307000.7<br>ENST00000553106.6	52.748713<br>16.981983
PCK1	phosphoenolpyruvate carboxykinase 1	ENSG00000124253	ENST00000319441.6	0.016257
PHLDA1	pleckstrin homology like domain family A member 1	ENSG00000139289	ENST00000602540.5<br>ENST00000266671.10	14.406729<br>0.606953
PLIN1	perilipin 1	ENSG00000166819	ENST00000430628.2	0.197608
PLSCR1	phospholipid scramblase 1	ENSG00000188313	ENST00000487389.5<br>ENST00000342435.9<br>ENST00000448787.6	9.816466<br>7.509352<br>1.71921
PON3	paraoxonase 3	ENSG00000105852	ENST00000265627.10<br>ENST00000451904.5	5.899761<br>0.263607
PRRG4	proline rich and Gla domain 4	ENSG00000135378	ENST00000257836.4	0.728434
RHOB	ras homolog family member B	ENSG00000143878	ENST00000272233.6	91.588906

RND3	Rho family GTPase 3	ENSG00000115963	ENST00000263895.9<br>ENST00000375734.6	28.855417<br>7.529461
RPP25L	ribonuclease P/MRP subunit p25 like	ENSG00000164967	ENST00000378959.9<br>ENST00000297613.4	12.579198<br>3.391974
SAA4	serum amyloid A4, constitutive	ENSG00000148965	ENST00000278222.7	1.164446
SAT2	spermidine/spermine N1-acetyltransferase family member 2	ENSG00000141504	ENST00000269298.10<br>ENST00000573566.1	50.874831<br>22.663125
SCARB1	scavenger receptor class B member 1	ENSG00000073060	ENST00000261693.11<br>ENST00000415380.6<br>ENST00000546215.5<br>ENST00000680556.1<br>ENST00000680596.1<br>ENST00000544327.1	91.542205<br>68.506383<br>59.199431<br>26.369478<br>6.578246<br>4.461932
SCD	stearoyl-CoA desaturase	ENSG00000099194	ENST00000370355.3	1147.411199
SDC2	syndecan 2	ENSG00000169439	ENST00000302190.9<br>ENST00000522911.5<br>ENST00000518385.5	39.821114<br>24.342346<br>11.471846
SERPINA1	serpin family A member 1 (α1-antitrypsin)	ENSG00000197249	ENST00000636712.1<br>ENST00000393087.9<br>ENST00000402629.1<br>ENST00000393088.8<br>ENST00000437397.5<br>ENST00000448921.5<br>ENST00000440909.5	2162.842686<br>19.373091<br>4.135652<br>1.256812<br>1.110108<br>0.346714<br>0.212755
SERPINH1	serpin family H member 1	ENSG00000149257	ENST00000358171.8<br>ENST00000533603.5<br>ENST00000524558.5<br>ENST00000530284.5	239.676154<br>11.505526<br>1.122409<br>0.133934
SFRP5	secreted frizzled related protein 5	ENSG00000120057	ENST00000266066.4	2.202643
SLC2A2	solute carrier family 2 member 2	ENSG00000163581	ENST00000314251.8	0.582367
SLC10A1	solute carrier family 10 member 1	ENSG00000100652	ND	0
SPTBN1	spectrin beta, non-erythrocytic 1	ENSG00000115306	ENST00000356805.9<br>ENST00000333896.5	171.825791<br>81.986192
SULT1A1	sulfotransferase family 1A member 1	ENSG00000196502	ENST00000314752.12<br>ENST00000569554.5	9.332073<br>0.184844
TAT	tyrosine aminotransferase	ENSG00000198650	ENST00000355962.5	2.269519
TF	transferrin	ENSG00000091513	ENST00000402696.9	195.691322
TFR2	transferrin receptor 2	ENSG00000106327	ENST00000462107.1	7.812334
TFRC	transferrin receptor	ENSG00000072274	ENST00000360110.9<br>ENST00000392396.7<br>ENST00000420415.5<br>ENST00000698290.1<br>ENST00000698285.1<br>ENST00000698291.1<br>ENST00000698295.1<br>ENST00000698280.1	468.574101<br>10.269163<br>2.467132<br>1.365116<br>1.115318<br>0.747771<br>0.57931<br>0.333346
TKFC	triokinase and FMN cyclase	ENSG00000149476	ENST00000394900.8	27.045395
TM4SF4	transmembrane 4 L six family member 4	ENSG00000169903	ENST00000305354.5	4.740282
TM4SF5	transmembrane 4 L six family member 5	ENSG00000142484	ENST00000270560.4	52.256383

TM7SF2	transmembrane 7 superfamily member 2	ENSG00000149809	ENST00000279263.14<br>ENST00000612081.1	118.745948<br>1.18996
TMEM97	transmembrane protein 97	ENSG00000109084	ENST00000226230.8<br>ENST00000336687.6<br>ENST00000582113.1	327.625775<br>37.141432<br>1.365351
TP53INP2	tumor protein p53 inducible nuclear protein 2	ENSG00000078804	ENST00000374810.8<br>ENST00000374809.6	23.691592<br>4.407257
TTC36	tetratricopeptide repeat domain 36	ENSG00000172425	ENST00000302783.10	0.134539
TTR	transthyretin	ENSG00000118271	ENST00000237014.8<br>ENST00000649620.1	333.165604<br>1.859561
TUBA1B	tubulin alpha 1b	ENSG00000123416	ENST00000336023.9	3272.391142
UCP2	uncoupling protein 2	ENSG00000175567	ENST00000663595.2<br>ENST00000536983.5	39.791085<br>1.472098
UGT1A1	UDP glucuronosyltransferase family 1 member A1	ENSG00000241635	ENST00000305208.10<br>ENST00000360418.4	1.281165<br>0.093469
VDR	vitamin D receptor	ENSG00000111424	ENST00000549336.6	0.622198
VIM	vimentin	ENSG00000026025	ENST00000224237.9<br>ENST00000339485.4	2006.517712<br>1.493837
WT1	WT1 transcription factor	ENSG00000184937	ND	0
WTAP	WT1 associated protein	ENSG00000146457	ENST00000337387.4<br>ENST00000621533.5<br>ENST00000614346.4<br>ENST00000631126.2	36.19295<br>24.856099<br>11.36496<br>2.759872
ZHX2	zinc fingers and homeoboxes 2	ENSG00000178764	ENST00000314393.6	26.416903
ACTB	actin beta	ENSG00000075624	ENST00000646664.1<br>ENST00000676397.1<br>ENST00000493945.6<br>ENST00000432588.6<br>ENST00000642480.2<br>ENST00000473257.3<br>ENST00000675515.1	3716.97217<br>33.690344<br>23.548124<br>6.407391<br>5.493343<br>2.582519<br>0.164191
GAPDH	glyceraldehyde-3-phosphate dehydrogenase	ENSG00000111640	ENST00000229239.10<br>ENST00000396856.5<br>ENST00000396859.5<br>ENST00000619601.1<br>ENST00000396861.5<br>ENST00000396858.5	5285.775364<br>31.631692<br>31.531195<br>16.213084<br>15.90503<br>0.202033

[1] Please note that, although the depicted genes are often described as hepatocyte-specific in various studies, many of these genes are also present in other liver cells. For example, the scavenger receptor class B member 1 (*SCARB1*), also referred to as SR-B1, is highly expressed in liver sinusoidal endothelial cells (Ganesan et al., 2016). [2] To compare the transcript levels of the listed genes, the expressions of actin beta (*ACTB*) and glyceraldehyde-3-phosphate dehydrogenase (*GAPDH*) are shown. The complete mRNA expression profile of HuH-7 cells observed by NGS can be found in Table S1. Abbreviations used: ND, no transcripts of this gene were detected; TPM, Transcripts Per Million.

Supplementary Figures

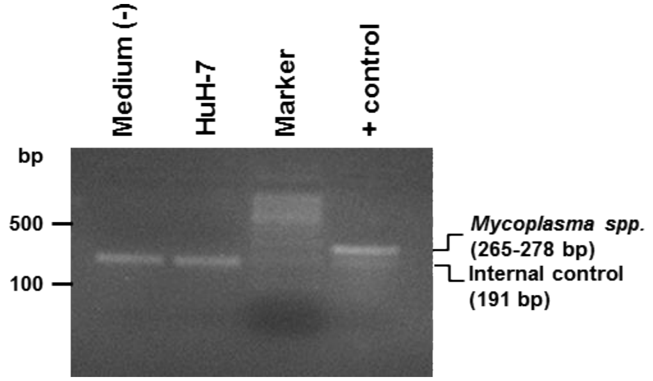

Supplementary Figure 1. Mycoplasma testing. In the depicted analysis, 2 µL of normal control medium (Medium (−)) and 2 µL of HuH-7 cell culture supernatant (HuH-7) were tested for mycoplasma infection. As a positive control, 2 µL of the positive control included in the chosen kit system was also co-amplified. The internal control produced a 191 bp fragment with each probe, indicating that the PCR reactions were functioning correctly. Amplification of a 265-278 bp fragment, as observed in the positive control sample, confirms the presence of a mycoplasma contaminant. The amplicons were separated on a 2 % standard agarose gel containing Midori Green, and then visualized using a standard gel imager.
